# Green synthesis of hyaluronic acid coated, thiolated chitosan nanoparticles for CD44 targeted delivery and sustained release of Cisplatin in cervical carcinoma

**DOI:** 10.3389/fphar.2022.1073004

**Published:** 2023-01-12

**Authors:** Kousain Kousar, Faiza Naseer, Maisa S. Abduh, Salik Kakar, Rabia Gul, Sadia Anjum, Tahir Ahmad

**Affiliations:** ^1^ Industrial Biotechnology, Atta-ur-Rahman School of Applied Biosciences, National University of Sciences and Technology, Islamabad, Pakistan; ^2^ Shifa College of Pharmaceutical Sciences, Shifa Tameer e Millat University, Islamabad, Pakistan; ^3^ Immune Responses in Different Diseases Research Group, Department of Medical Laboratory Sciences, Faculty of Applied Medical Sciences, King Abdulaziz University, Jeddah, Saudi Arabia; ^4^ School of Health Sciences, National University of Sciences and Technology, Islamabad, Pakistan; ^5^ Department of Biology, University of Hail, Hail, Saudia Arabia

**Keywords:** polymeric nano composites, cervical cancer, Cisplatin, CD44, thiolated chitosan, sustained realease, hyaluronic acid, targeted delivery

## Abstract

Cervical carcinoma is one of the most prevalent gynecological cancers throughout the world. Cisplatin is used as first line chemotherapy for treatment of cervical cancer, but it comes with plethora of side effects. The aim of this study was to develop hyaluronic acid coated, thiolated chitosan nanocarriers using green synthesis approach, for CD44 targeted delivery and sustained release of Cisplatin in cervical cancer cells. After synthesis through ionic gelation method, Zeta analysis showed that the nanoparticle size was 265.9 nm with a zeta potential of +22.3 mV and .226 PDI. SEM and TEM analysis confirmed the spherical shape and smooth surface of nanoparticles. FTIR and XRD showed the presence of characteristic functional groups, successful encapsulation of drug, and crystalline nature of nanoparticles respectively. Drug loading and entrapment efficiency were calculated to be 70.1% **±** 1.2% and 45% ± .28% respectively. Analysis of *in vitro* drug release kinetics showed that drug release followed the Higuchi model at pH 6.8 and 7.4 and Cisplatin release for up to 72 h confirmed sustained release. *In vitro* analysis on cervical cancer cells HeLa and normal cervical epithelial cells HCK1T was done through cell morphology analysis, trypan blue assay (concentration range of 10–80 μg/ml), and MTT cytotoxic assay (concentration range of 10–90 μg/ml). The results showed a higher cytotoxic potential of HA coated, thiolated chitosan encapsulated Cisplatin (HA-ThCs-Cis NP) nanoformulation as compared to pure Cisplatin in HeLa while in HCK1T, pure Cisplatin showed much higher toxicity as compared to HA-ThCs-Cis nanoformulation. These findings suggest that CD44 targeted delivery system can be a useful approach to minimize offtarget toxicities, give sustained release and better cellular uptake in cancer cells.

## Graphical Abstract

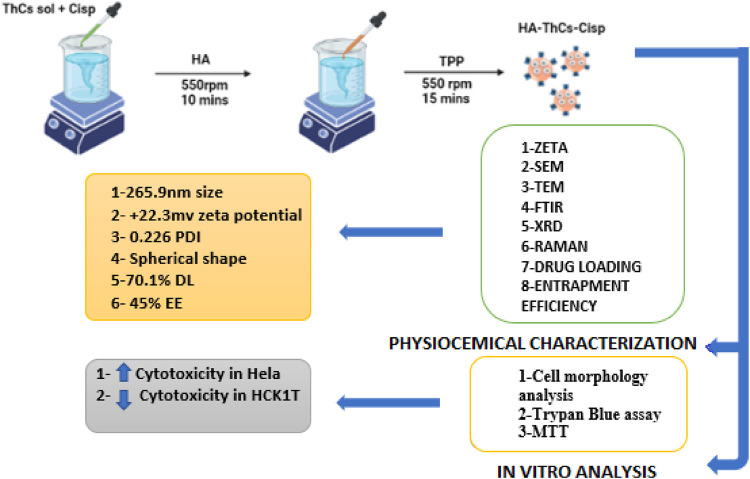

## 1 Introduction

Cervical cancer (CC) or carcinoma of the cervix is the fourth most common cancer of women in the world and claim lives of approximately 273,000 women every year (Globocan Cancer Observatory, 2020). Human Papilloma virus (HPV) infection accounts for the major risk factor associated with stimulation of oncogenic transformation of the cervix (Ulrikh, 2020). The current treatment modalities of CC include intravenous administration of Cisplatin (Cis) along with radiation therapy, followed by partial or complete hysterectomy (Wang & Liang, 2020). Cis acts by interfering with DNA replication and transcription mechanisms, by stimulating specific signal transduction pathways such as mitochondrial signaling pathway, death receptor signaling and calcium signaling to induce apoptosis in cells without distinguishing cancer and normal cells and therefore cause severe off-target toxicities (Aldossary, 2019). To minimize these toxicities, a targeted drug delivery system can be used to deliver Cis to cancer cells only.

Biocompatible, polymeric nanoparticles-based multifunctional drug delivery systems (DDS) have emerged as potential nanoparticles (NPs) with the flexibility of surface modification. These DDS can be customized for both active and passive targeting of tumor tissues. Surface functionalization with specific targeting moieties facilitate the binding and cellular uptake through specific receptors overexpressed on cancer cells only (Gagliardi et al., 2021). Chitosan (D-glucosamine and N-acetyl-D-glucosamine) obtained by partial deacetylation of chitin, is a linear cationic polymer connected through linear β-(1→4) glycosidic bonds. Also, it is approved by United States Food and Drug Administration (US FDA) for wound healing purpose and is rendered as GRAS (Generally Recognized as Safe) by US FDA. Owing to charged active amine groups (+NH2), the structure of chitosan is flexible for physical modifications (Nayak et al., 2022). Thiolated chitosan (ThCs) is obtained by covalent binding of various –SH group bearing moieties mainly with primary the amino group or with hydroxyl the group of chitosan polymer. ThCs has the potential of modification for targeted interaction at the site of tumor cells due to the presence of a covalently bound free thiol group. ThCs-based nanoparticles can interact with the surface of cellular membrane through electrostatic forces of attraction, physical mechanisms, and non-covalent binding (Federer et al., 2020). The characteristic flexibility of modification of chitosan can be utilized to form nano delivery system by physically crosslinking with anionic molecules like oxalate, tripolyphosphate, sulfate and citrate in chains of another polymer through the formation of ionic or covalent bonds (Garg et al., 2019).

Ionic gelation method, which is based on ionic crosslinking or electrostatic forces of attraction between oppositely charged groups, is one of simplest method to formulate nano DDS (Buyuk et al., 2020). It involves reversible physical crosslinking, avoids the use of harsh chemicals, organic solvents and does not require much apparatus and time. An anionic cross linker like tripolyphosphate polyanion (TPP) can be used to crosslink chitosan chains into a closed drug delivery system, through interaction between abundant NH_2_ group (amine) on chitosan and negatively charged phosphoric ions of TPP (Al-nemrawi et al., 2018) In cancer targeting, ThCs nanoparticles have the following advantages 1) Increased mucoadhesion due to greater amount of amidine moieties which facilitate binding and retention at the cellular membrane. 2) Better cohesive properties due to the presence of the intramolecular or intermolecular disulfide bond. 3) Improved permeation as ThCs-NPs can overcome electric resistivity and loosen the cellular tight junctions. 4) Increased bioavailability of drug due to enhanced permeation and retention effect. 5) Targeting ligand on the surface facilitates binding and uptake through specific receptor-mediated endocytosis (Jain et al., 2022).

Hyaluronic acid is a naturally occurring, non-sulfated, hydrophilic, negatively charged mucopolysaccharide consisting of d-glucuronic acid and N-acetyl-d-glucosamine units. Depending upon the length of the chain, its molecular weight ranges from thousand to million Daltons and determines its functional properties. It has promising therapeutic applications because it can undergo many conformational changes, can conjugate with compatible ligands, crosslink bioactive compounds due to the presence of N- acetyl groups, and has primary and secondary hydroxyl groups, glucuronic acid, and carboxylic acid groups at functionally reactive sites. Surface functionalization of nano DDS with hyaluronic acid (HA) has benefits of increased blood circulation time, enhanced biocompatibility, and active targeting of CD44 receptors on cancer cells (Naseer et al., 2011). CD44, also known as H-CAM is a stem-like a receptor that has been found to be over-expressed on solid cancers like prostate cancer, breast cancer, glioblastoma, colon cancer, and cervical cancer. This receptor has been implicated in various physiological processes pivotal to carcinogenesis such as drug resistance, tumour progression, invasion, and metastasis. HA/CD44 interaction is central to the invasion which is why cancer cells express these receptors greatly (Zhong et al., 2016). High CD44 expression in HPV-16 positive cervical cancer has been associated with increased metastasis, high colony formation capacity, and resistance against radiation therapy. A study reported 86% surface expression of CD44 receptor on HeLa, CasKi and INBL cervical cancer cell lines (Gutiérrez-Hoya et al., 2019). CD44/HA has high affinity and upon binding, the receptor/ligand complex is internalized through the caveolin/clathrin mediated endocytosis pathway which leads to higher uptake and retention inside the cell (Kesharwani et al., 2022).

**FIGURE 1:**
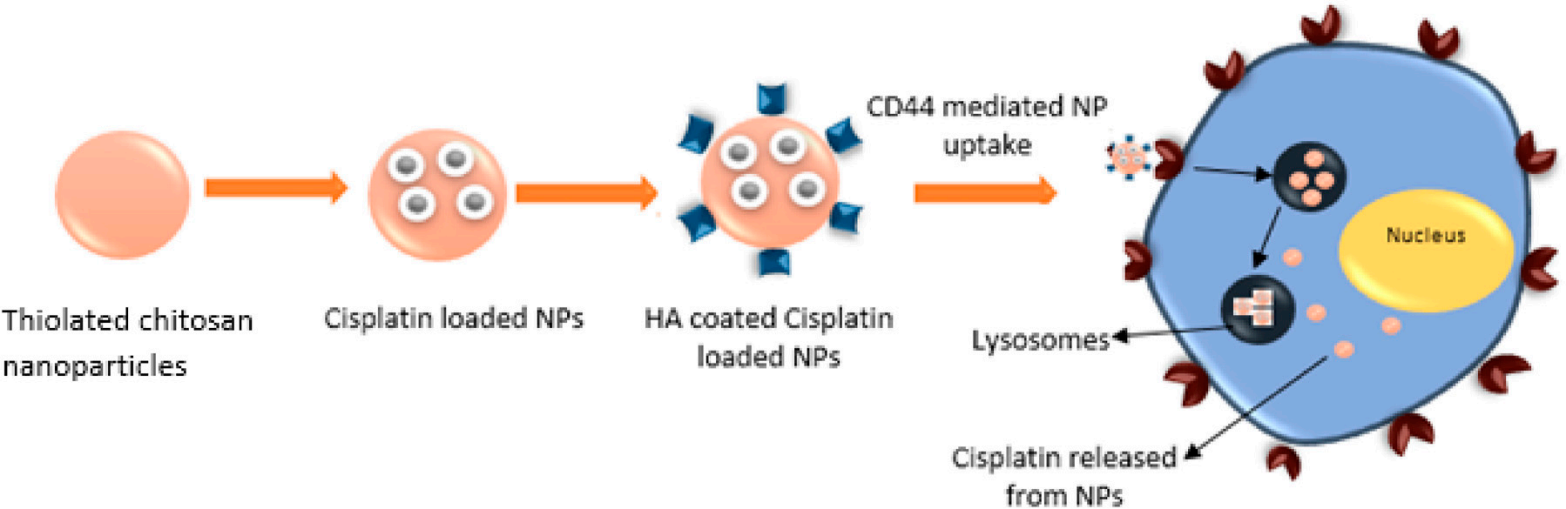
HA functionalized, ThCs nanoformulation for CD44 targeted delivery and sustained release of drug in cancer cell.

The rationale of the study was to analyze the efficacy of HA targeted, ThCs nanodrug delivery system in decreasing Cis associated toxicities and sustained release of drug. The aim of this research was to formulate a ThCs based nano drug delivery system (DDS), surface functionalized with HA for CD44 targeted delivery of Cis inside cervical cancer cells. As this formulation is based on polymers extracted from natural sources and no use of acids/harsh chemicals or hazardous by-products were involved in formulation process, so it comes under the umbrella of green synthesis. This CD44 targeting leads to nanoparticle internalization by receptor-mediated endocytosis for enhanced uptake, followed by drug release inside the cell as shown in Figure 1 below:

## 2 Materials and methods

### 2.1 Materials

Chitosan (Cs) low molecular weight (50–190 kd) with 75%–80% deacetylation, Tripolyphosphate polyanions (TPP) and Thioglycolic acid (TGA) was obtained from Quaid-e-Azam University, Islamabad, Pakistan. Glacial acetic acid, Sodium dihydrogen phosphate, potassium dihydrogen phosphate, dipotassium hydrogen phosphate, 1-ethyl-3-3 (3-dimethylamino propyl carbodiimide hydrochloride (EDC), sodium hydroxide hydroxlamine, dialysis membrane of high retention capacity (12,000–14,000 Mw cut-off), hydrochloric acid (HA), and sodium borohydride, were purchased from Sigma-Aldrich United States by Biolife Technologies and Science Home Traders. Cell culture media DMEM, fetal bovine serum and Pan-strap were purchased from Gibco through Biolife technologies Islamabad. Ellman’s reagent [5,5′-Dithiobis (2-nitrobenzoic acid; DTNB)] and N-hydroxy succinimde (NHS) were purchased from Germany. All chemicals and solvents used in the experiment were of analytical grade.

### 2.2 Molecular docking for HA/CD44 binding

Molecular docking of hyaluronic acid with CD44 receptors was performed to determine the *in silico* binding affinity of the hyaluronic acid with CD44 receptor protein.

The crystal structure of the protein was obtained from the Protein data bank (PDB.com) for CD44 (PDB ID: 4PZ3) and Pubchem was used to obtain structure of Hyaluronic acid (PubChem CID: 23663392). PyMOL 1.8 was used for the manual evaluation of the protein structure and cleaning of proteins, PyRX was used for the molecular docking study, The binding domains of proteins were identified by using a database called CASTp. Discovery studio 2017 was used for the visualization of the docking results and interactions (Kondapuram et al., 2021).

### 2.3 Preparation of thiolated Chitosan

**FIGURE 2:**
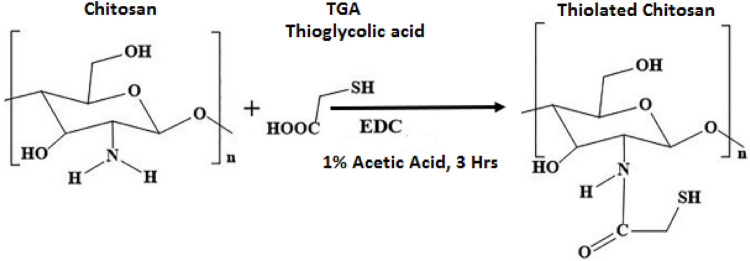
Reaction scheme of ThCs synthesis by formation of an amide bond between the carboxyl group of thioglycolic acid and amine group of chitosan in presence of EDC.

To prepare ThCs, 1% chitosan solution was prepared in 1% acetic acid solution. To this solution, 6.9 ml of thioglycolic acid or TGA and 50 mM of EDC was added which initiated the process of amide bond formation between chitosan and TGA through activation of carboxylic groups on TGA, as shown in Figure 2. Hydroxylamine solution was added to prevent oxidation during the process. The pH of the mixture was adjusted to 5.8 using 1M NaOH. This mixture was dialyzed continuously for 3 days with constant stirring to remove any unbound TGA. The dialyzed medium (5 L of 5 mM solution of HCL) was refreshed after every 6 h, followed by a 5 L of 5 mM HCl with 1% NaCl for next 6 h and last 6 h with the 5 L of 5 mM solution of HCL again. The resulting solution was stored at −80°C for 2 days before lyophilization (Alpha 1–2 LD plus, Germany). After lyophilization, white amorphous material was obtained which was stored at 4°C until further use (Hasanifard et al., 2017).

### 2.4 Estimation of thiol group using Ellman’s reagent

Spectrophotometry based analysis was done to assess the degree of thiol group substitution in ThCs polymer. For this purpose, 5, 5′-dithiobis (2-nitrobenzoate) (DTNB) also called Ellman’s reagent was used which is a hydrophilic, symmetric aryl sulfide compound. 5 mg of ThCs was dissolved in ultrapure deionized water, after dissolution of ThCs, .25 ml of phosphate buffer was added while maintaining pH at 8. This was followed by the addition of .5 ml of Ellman’s reagent and the resulting solution was incubated for 2 h at 25°C. After 2 h this polymeric suspension was centrifuged at 23,700 rpm for 10 min, the supernatant was collected, and absorbance was measured using spectrophotometer at 420 nm wavelength. The control samples carried non-thiolated chitosan. From the corresponding control, the number of thiol groups were calculated, and a standard curve was obtained against the TGA standards (Saboktakin, 2017).

### 2.5 Preparation of HA-coated ThCs blank nanoparticle

**FIGURE 3:**
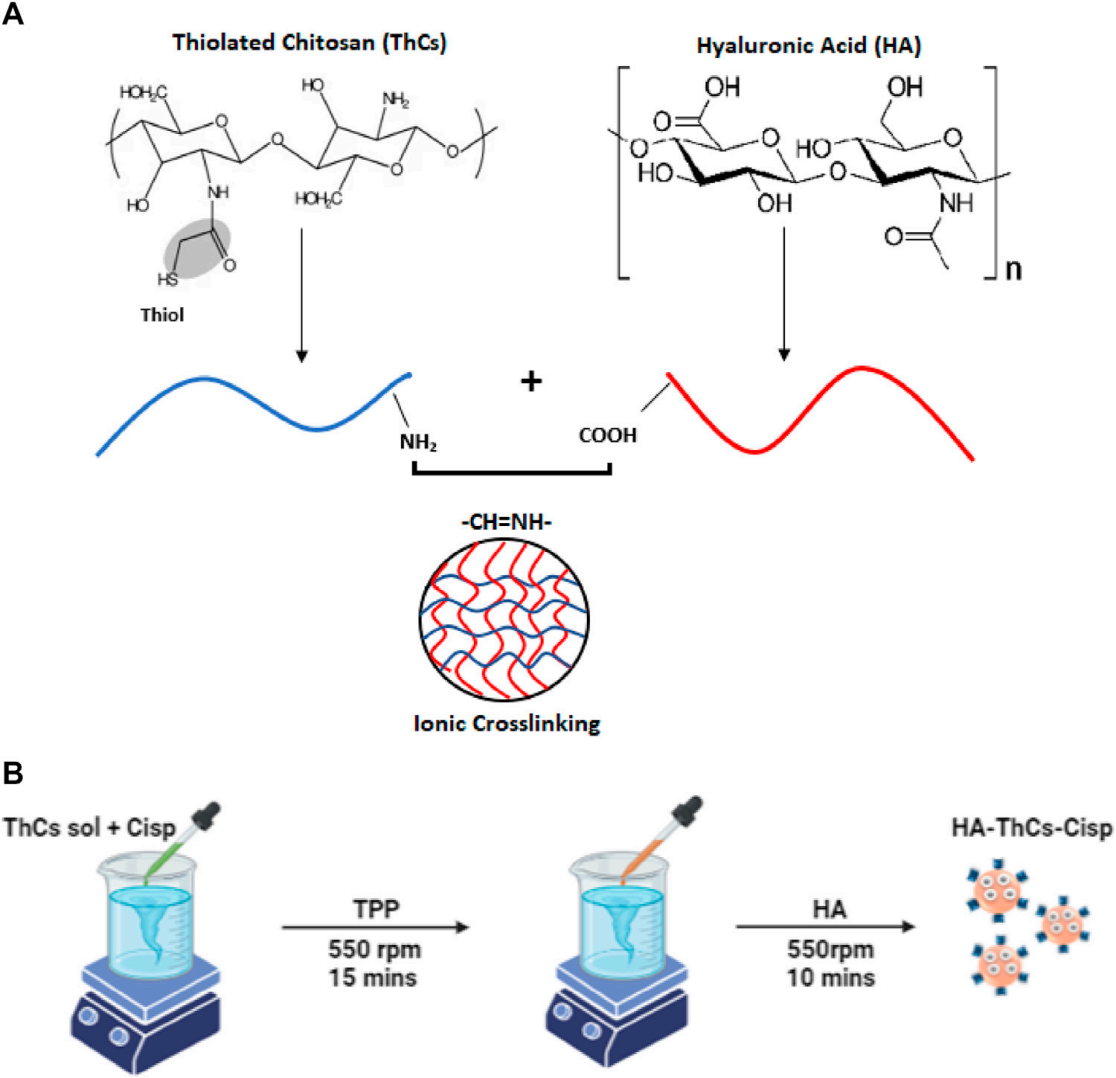
**(A)** Ionic cross linking between oppositely charged moieties of Cs and HA during ionic gelation process. **(B)** Preparation of HA-ThCs-Cis nanoparticles using Ionotropic Gelation method.

HA coated ThCs nanoparticles were prepared using a beaker, magnetic stirrer, dropper and other simple equipment. The formulation was based on principle of ionic crosslinking in which nanoparticles were formed due to the electrostatic forces of attraction between NH_2_ group of chitosan and COOH group of hyaluronic acid as shown in Figure 3A. To a solution of ThCs, TPP was added drop wise as polylinker at concentration of .1 mg/ml with constant stirring to allow formation of empty/blank ThCs nanoparticle. This step was followed by addition of HA solution to allow its attachment on surface of ThCs nanoparticles as shown in Figure 3B (Wang et al., 2016).

### 2.6 Preparation of Cis loaded HA-ThCs nanoparticles

With a slight modification in above reaction, Cis loaded nanoparticles were prepared. For nanoparticle optimization, solutions of ThCs were prepared at different concentration (.10–1 mg/ml of chitosan) in distilled water. In a solution of ThCs, .15 mg/ml of Cis was added drop wise through a syringe while stirring constantly at 530 rpm for 10 min. This was followed by addition of .10 ml of cross linker TPP to the solution with constant stirring at 530 rpm for 15 min which resulted in ThCs nanoformulation of different concentrations. All NPs formulations were sonicated at 30 mA for 15 min for purpose of nanoparticles dispersion and homogenization. After sonication, drug loaded nanoparticles were coated with HA as described earlier, centrifuged, lyophilized, and stored at 4°C until further use (Sultan et al., 2022). Schematic diagram Figure 3B shows the preparation procedure.

### 2.7 Optimization of NPs formulation preparation

Box Behnken factorial design is a systematic tool to optimize a system in short period of time. To synthesize a highly accurate nano drug delivery system, Box-Behnken design (BBD) was used for optimization of HA-ThCs-Cisplatin nanoparticles employing Design of Expert Software (DOE version 8.0.6.) The independent variables included concentration of chitosan, Cisplatin and hyaluronic acid, while the dependent variables included nanoparticle size, PDI, zeta potential and encapsulation efficiency. The best optimized formulation was finally selected and further characterized for different physiochemical parameters (Akram & Garud, 2021).

### 2.8 NPs formulation physiochemical characterization

Characterization plays a significant role in evaluating the potential of the NPs formulation for targeted delivery and uptake at the site of tumor.

#### 2.8.1 SEM analysis

SEM (Scanning electron microscopy) analysis was done using MIRA3 (TESCAN, Czech Republic) to observe the microstructure and surface chemistry of blank and drug-loaded HA-ThCs NPs formulation. A small amount of lyophilized powder was fixed on aluminium stubs followed by coating with gold. SEM images of nanoparticles were taken using accelerated electrons with 15 kV voltage to image samples at nanometer scale resolution (Khouchaf and Oufakir, 2022).

#### 2.8.2 TEM analysis

Transmission electron microscopy (TEM) [with (JEOL/JEM 2100, Akishima, Tokyo, Japan)] was used for analysis of NPs to determine size, shape, and confirm drug loading in nanoparticles. A single drop of blank HA-ThCs and HA-ThCs-Cis was dried on a copper grid and images were taken by operating at a voltage of 200 kV (Khouchaf and Oufakir, 2022).

#### 2.8.3 Physiochemical analysis of NPs formulation

Using Malvern Zetasizer Nanos ZS90 (Malvern Instruments, United Kingdom) zeta potential, average particle size, and PDI of blank and Cis loaded HA-ThCs was measured. Zeta Sizer gives hydrodynamic diameter of particles, readings of diluted nanoformulation (1:10) were taken at 25°C. Three different batches were evaluated on a zeta nanosizer to obtain an average value and to calculate the standard deviation for zeta potential, average size, and PDI. NPs PDI is a ratio of weight to number-average molecular mass (Doğan, 2020).

$$PDI=\frac{M_{w}}{M_{n}}$$

#### 2.8.4 Raman spectroscopic analysis

Raman Instrument from Thermo Fisher Co., Ltd., (United States) was used to examine vibrational, rotational, and other low-frequency modes of molecules in blank and drug-loaded HA-ThCs NPs. Raman shift was observed using 150 mW laser power and at the wavelength range of 0–3,500 cm^−1^ and 780 nm excitation wavelength. The same parameters were used for the collection of all spectral data.

#### 2.8.5 X-ray diffraction (XRD) study

X-ray diffraction and scattering (XRD) of blank and drug-loaded HA-ThCs was analyzed at room temperature, by using a D8 ADVANCE X-Ray diffractometer (Bruker, Germany). This was done to evaluate the crystalline nature of NPs formulation. The resulting patterns of XRD were collected at an angular range of 10°–50° with 2θ in continuous mode using a step size of .03 2θ and step time of 60 s.

#### 2.8.6 Functional group identification of NF

For functional group determination and confirmation of successful encapsulation of Cis inside NPs, FTIR analysis was done using an FTIR spectrometer (PerkinElmer, Waltham, MA, United States) (Mythili, 2017). The characteristics peaks of functional groups were determined using lyophilized powder of blank and Cis-loaded nanoparticles.

#### 2.8.7 Drug loading and encapsulation efficiency

Drug content in the supernatant is the indirect measure of the drug loaded in nanoparticles and was used to determine the amount of Cis loaded in ThCs nanoparticles, and to calculate the encapsulation efficiency of NPs formulation. For this purpose, NPs formulation was centrifuged for 1 h at 13,700 rpm, followed by the collection of supernatant. This supernatant was filtered using a syringe filter and tested for concentration of free drug at 269 nm using a UV-Vis spectrophotometer (NanoDrop 2000c; Thermo Scientific, Wilmington, DE, United States). Following formulas were used for calculation of drug loading (DL) and encapsulation efficiency (EE) (Gadkari et al., 2019).

Indirect method for calculation of DL and EE:

• % Drug loading (DL) = (W_1_−W_2_) × 100

$$W_{3}$$

Where {W_1_= Total Cis added, W_2_ = Cis in supernatant, W_3=_ Amount of ThCs (Polymer)}

• % Encapsulation efficiency (EE) = (W_1_−W_2_) × 100

$$W_{1}$$

Where {W_1_ = Total amount of Cis and W_2_ = Free Cis in supernatant}

#### 2.8.8 Calibration curve of Cis in distilled water

One milligram of Cis was dissolved in 50 ml of distilled water according to BP guidelines to prepare calibration curve of the anticancer drug Cis. Using different concentrations of the drug ranging from .1 to 1 mg/ml, different dilutions of the drug were prepared in distilled water. The distilled water was used as blank, and all dilutions of the drug were analyzed at 269 nm using a UV-Vis spectrophotometer. A calibration curve was plotted using MS-Excel and it was used for the calculation of unknown concentrations of Cis in NPs formulation samples (Sultan et al., 2022).

#### 2.8.9 Calibration curve of Cis in phosphate buffer 7.4 pH

A calibration curve was drawn to determine the concentration of Cis in the release medium. For this purpose, 1 mg of Cis was dissolved in 10 ml of phosphate buffer at 7.4 pH. This solution was then sonicated for 10 min to allow complete dissolution. As described above for the calibration curve of Cis in distilled water, serial dilutions were prepared from this solution following sonication. Phosphate buffer was used as a reference and all dilutions of the drug were analyzed at 269 nm using a UV-Vis spectrophotometer. A calibration curve was plotted using Graphpad Prism 9 and it was used for the calculation of *ex vivo* and *in vitro* drug release profiles.

### 2.9 Evaluation of *in-vitro* drug release from NPs

To determine the release of Cis from NPs, a drug release assay was conducted by immersing a dialysis membrane bag in phosphate buffer at pH 7.4 and 6.8 while keeping temperature at 37°C. Five milligram of Cis loaded NPs lyophilized powder was added to 50 ml of phosphate buffer at both pH in a dialysis bag, this dissolution medium was constantly stirred at 100 RPM on a magnetic hot plate. After specific time intervals of 0, 1, 2, 3, 4, 7, 12, 24, 48, and 72 h, 1.5 ml of sample was collected and analyzed at 269 nm using UV-Vis spectrophotometer (NanoDrop 2000c; Thermo Scientific, Wilmington, DE, United States). A standard calibration curve of Cis in phosphate buffer at pH 6.8 and 7.4 was created on MS-Excel sheet using results calculated by the following formula. The study was conducted on triplicate samples

$$In-vitro\;drug\;released\;\left(\%\right):\;\frac{Cis\;\left(drug\right)\;released\;in\;buffer\times 100}{Total\;Cis\;\left(drug\right)\;added}$$

### 2.10 Drug release kinetics

The therapeutic efficacy of the drug depends on the release mechanism of the drug from the nano formulation. Various kinetic models were applied to evaluate the permeability and *in vitro* release of drugs to understand the mechanism of drug release from NPs.

#### 2.10.1 Zero order drug release kinetics

Zero-order kinetics describe that a drug (Cis) is released from the nanocarrier at a constant rate. To evaluate Cis release kinetics according to zero order, a graph was plotted between cumulative drug releases versus time. In zero-order kinetics, the rate of drug release per unit of time is independent of the concentration of a drug (Herdiana et al., 2022). The slope of the graph represents zero order release constant and can be represented by the following equation:

W=k1t

Where {k1 = zero order release constant; W = cumulative drug release; t = time in hours}

#### 2.10.2 First order drug release kinetics

First order kinetics also describe the amount of Cis released per unit time but unlike zero order kinetics it is dependent on the concentration of the drug (Craciun et al., 2019). The graph was plotted between log of cumulative drug release versus time and represented by following equation:

$$\ln\left(100-W\right)=\ln100\;–\;k2t$$

Where {W = cumulative drug release; k2 = first order release constant}

#### 2.10.3 Higuchi square root of time equation (diffusion model)

Higuchi model describes the release of drug from matrix through diffusion in non-erodible manner (Pourtalebi Jahromi et al., 2020). The cumulative release of Cis was plotted against square root of time. Higuchi model expression is given by following equation:

$$W=k_{4}t$$

Where {W= cumulative drug release in time t; k4= Higuchi dissolution rate constant}

#### 2.10.4 Hixon Crowel’s cube root equation (erosion model)

The Hixon Crowel’s model explains release of drug from matrix system by erosion or drug release by dissolution due to changes in surface area and diameter of the drug particles. It describes drug release as erosion followed by diffusion (Herdiana et al., 2022). This model was illustrated by following equation:

$${\left(100\right)}^{1/3}\;100^{1/3}-W=-k_{3}t$$

where {k2 = Hixon release constant; W = cumulative drug release in time t through dissolution}

#### 2.10.5 Korsmeyer Peppas or power law equation (diffusion/relaxation model)

This model describes the release of drugs from the polymeric system. It simultaneously emphasizes on several release mechanisms such as diffusion of water in nanoparticle matrix, matrix swelling, and dissolution of matrix (Pourtalebi Jahromi et al., 2020). This model describes drug release vs. time with an exponential function and is represented by the following equation:

Mt/M∞=k5tn

Where {Mt/M∞ = Percent of drug release at time t; n = diffusion exponent for drug release; k5 = constant incorporates structural and geometric characteristics of sustained drug delivery system, n = exponential which characterize the mechanism of drug release}

(Value of n = .45 describes fickian diffusion and .45 < n > .9 illustrates non-fickian diffusion. n > .89 explains super case II release while n = .89 describes case II or zero-order release).

### 2.11 *In vitro* anticancer activity of NFs of Cis loaded in HA-ThCs

#### 2.11.1 Cell culture

The *in vitro* cytotoxic potential of Cis-loaded HA-ThCs was determined on the cervical cancer cell line (HeLa) and normal cervical epithelial cells HCK1T. In comparison to Cis-loaded HA-ThCs, cell lines were treated with the commercially available drug Cis at the same concentrations. HeLa and HCK1T cells were kindly provided by Dr. Madiha Zeeshan, Assistant Professor, Public Health and Nutrition department, University of Haripur, Pakistan, who obtained these cell lines from National Institute of Health (NIH), Islamabad, Pakistan. Both cell lines were cultured in DMEM, supplemented with 10% FBS (Gibco) and 1% pen/strep (100 units of penicillin, 100 µg of streptomycin) (Gibco). The cells were placed in a humidified atmosphere of 95% air and 5% CO_2_ at 37°C. The cells were grown in 75 cm^2^ tissue culture flasks and were used for the experiment after they reached the exponential growth phase (Jalilian et al., 2019).

#### 2.11.2 *In vitro* experimental groups

Three groups were formed for *in-vitro* experiment:

Group 1: Untreated control HeLa cells in DMEM medium.
Group 2: Cis treated HeLa cells.
Group 3: Cis loaded HA-ThCs NPs treated HeLa cells.
Group 4: Untreated control HCK1T cells in DMEM medium.
Group 5: Cis treated HCK1T cells.
Group 6: Cis loaded HA-ThCs NPs treated HCK1T cells.

#### 2.11.3 Cell morphology assay

Morphological features like membrane blobbing, shrinkage, nuclear and cytoplasmic condensation, and rounding of cells are the hallmark of apoptosis. To record these changes, HeLa and HCK1T cells were plated in a 96-well plate at a density of 1 × 10^6^ (100 µl) per well. After 24 h, media was refreshed and Cis -or Cis loaded HA-ThCs NPs were added to cells at different concentrations (10, 40, 80 μg/ml dissolved in phosphate buffered). Cell morphology was assessed using a phase contrast inverted microscope after 12 and 24 h to observe the morphological changes induced by nanoparticles and drug.

#### 2.11.4 Trypan blue exclusion assay

Trypan blue exclusion assay was performed to determine the number of living cells present in a suspension of treated cells. For this purpose, cells were seeded in a 24-well plate at a density of 130,000 cells/well in 1 ml. After 24 h, the supernatant was removed, and cells were detached and harvested using Trypsin-EDTA (Gibco, New York) for 5 min at 37°C. This was followed by the addition of a growth medium to stop the trypsin reaction and the cell palette was collected by centrifugation at 800 rpm for 3:30 min. Cells were washed with PBS (Gibco) and resuspended in 1 mL of fresh media. 50 µl of .4% trypan blue (Gibco, United States) was mixed with 50 µl of cell suspension and incubated for 3 min at 37°C. The mixture was then transferred to a hemacytometer for counting of viable (white) and dead cells (blue) under a phase contrast inverted microscope. The counting of cells should begin within 5 min of mixing cells with dye. The cell viability was calculated using the formula.

$$\%\;Cell\;viability=\frac{Viable\;cell\;count\;x\;100}{Total\;number\;of\;cells}$$

#### 2.11.5 MTT assay

MTT assay is a colorimetric assay based on the ability of the live cells to convert a water-soluble dye [3-(4,5 dimethylthiazol-2-yl)-2,5-diphenyltetrazolium bromide] into insoluble formazan by dehydrogenases in mitochondria of the living cells. The cytotoxicity of Cis-loaded HA-ThCs nanoparticles was evaluated through an MTT assay. HeLa and HCK1T cells were plated in a 96-well plate at a seeding density of 1 × 10^6^ (100 µl) per well. After 24 h media was refreshed and 100 µl of Cis and HA-ThCs-Cis at concentrations 80, 50, and 10 mg/ml dissolved in PBS was added. Cells were then incubated with 95% air and 5% CO_2_ at 37°C for 24 h, control cells were treated with PBS only at 7.4 pH. After 24 h, 10 µl of MTT dye solution was added to achieve a final concentration of .45 mg/ml. Cells were again incubated at 37°C and 5% CO_2_ for 4 h. After incubation, MTT dye was removed and 100 µl of DMSO was added to dissolve formazan crystal and the cells were incubated again for 1 h. The concentration of formazan crystal formed is directly proportional to the number of viable cells. The resulting-colored solution was quantified by using a microplate reader at 560 nm (BioTek, Winooski, VT, United States). Cell viability percentage was calculated using the following formula (Jalilian et al., 2019).

$$Cell\;viability\;\left(\%\right)=\frac{OD\;of\;Cells\;treated\;with\;drug\;/\;NPs-OD\;blank\times 100}{OD\;of\;control\;cells-OD\;of\;blank}$$

### 2.12 Stability evaluation

To analyze the stability of NPs formulation in terms of size, zeta potential, PDI, and shape, the particles were analyzed after 3 months while they were kept at 37°C or 4°C in lyophilized form. The nanoformulation was redistributed in ionized water before analysis.

### 2.13 Statistical analysis

The results of all studies were statistically analyzed by using one-way analysis of variance (ANOVA). A Fisher’s least significant differences *post hoc* analysis was performed, using an alpha value < .05 The results were reported as the mean of three samples (*n* = 3) along with the standard deviation (mean ± SD).

## 3 Results

### 3.1 Molecular docking of Hyaluronic acid with 4PZ3 (CD44)

**FIGURE 4:**
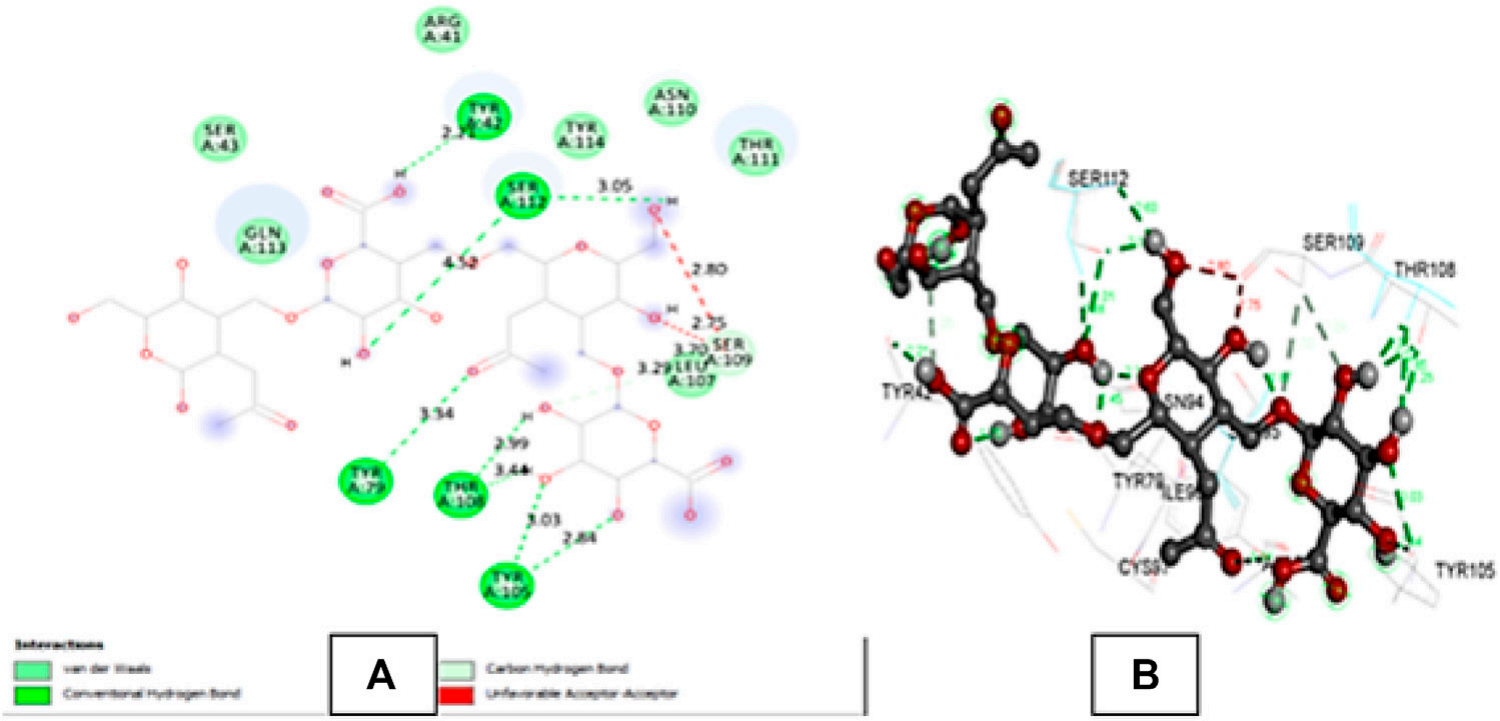
**(A)** shows the 2D interactions of Hyaluronic acid with the amino acid residues of 4PZ3 protein chain A and **(B)** shows the 3D interaction of Hyaluronic acid with the amino acid residues of the protein.

4PZ3 is a crystal structure consisting of two identical chains A and B (Liu & Finzel, 2014). The docking of hyaluronic acid with 4PZ3 (CD44 protein receptor) showed many hydrogen bonds in the green dotted lines in Figure 4 and Supplementary Table S1. All binding distances were in the acceptable range except 4.52 A°, and the interaction with SER A: 112. The ligand molecule also showed some unfavourable acceptor-acceptor interactions with SERA: 109 at 2.80 and 2.75 A° indicating some repulsion between the atoms. But overall, the binding affinity (−7.2) and interactions were strong.

### 3.2 Optimization of NPs formulation

**FIGURE 5:**
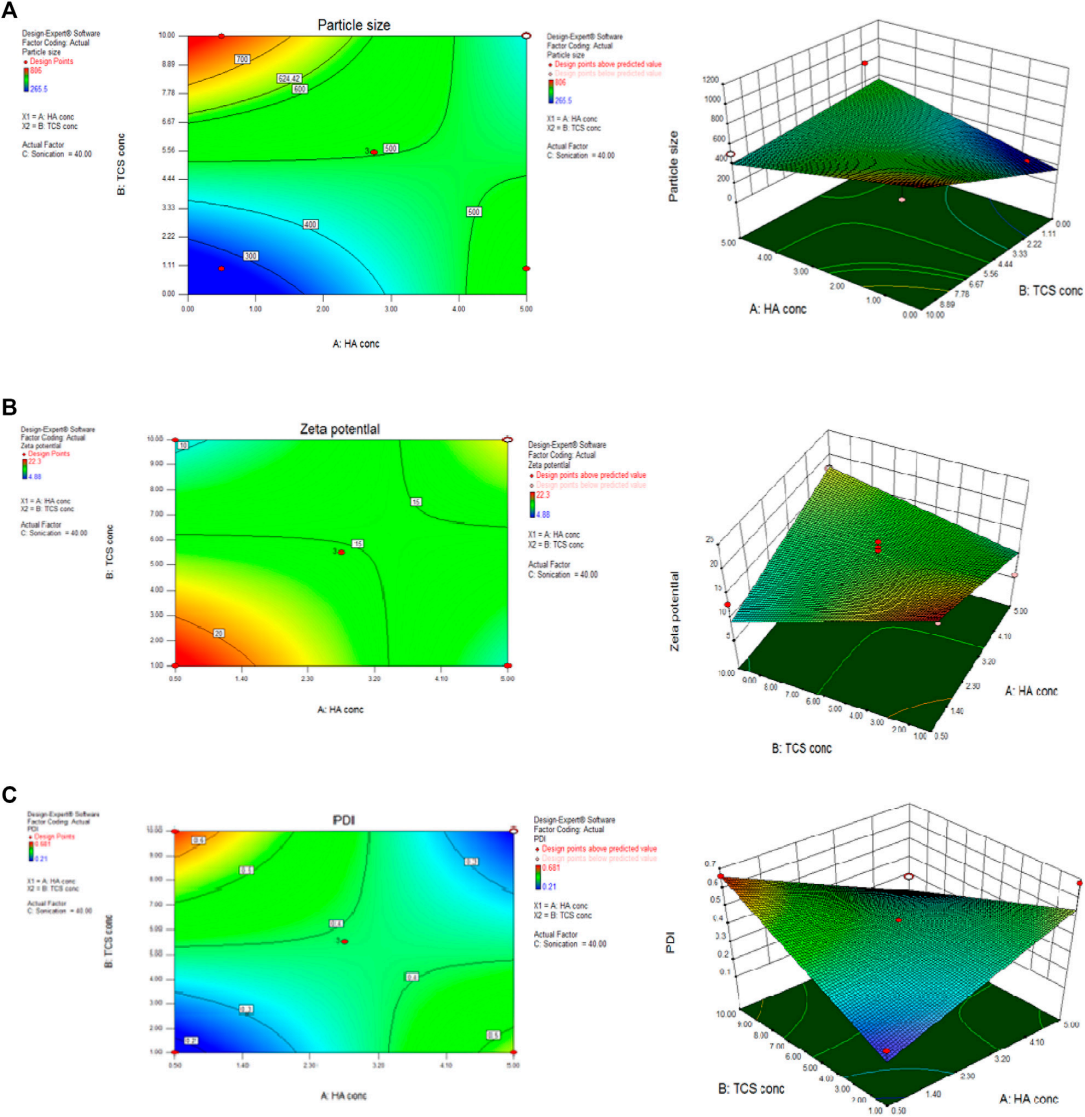
BoxBehnken factorial design by using Design of expert considering parameters of particle size **(A)** zeta potential **(B)** and PDI **(C)** to optimize NPs formulation.

The central composite design was chosen with the help of Design of Expert version 8.0.6.1 by following BoxBehken factorial design as shown in Figure 5. The measured dependent variables included nanoparticle size (Figure 5A), zeta potential (Figure 5B), polydispersity index (PDI) (Figure 5C), and encapsulation efficiency (EE) of NPs formulation. Formulation number 4 (Supplementary Table S2) was chosen for further evaluation.

### 3.3 Determination of thiol group

The degree of thiolation of chitosan polymer was calculated to be 787 μmol/G by using Ellman’s reagent. ThCs appeared as fibrous, white, odourless structure in lyophilized form which was aqueous soluble.

### 3.4 Characterization of NPs formulation

Detailed characterization of Cis loaded and blank HA-ThCs nanoparticles was carried out to assess their physiochemical properties and characteristic morphology.

#### 3.4.1 Physiochemical characteristics of HA-ThCs nanoparticles

**FIGURE 6:**
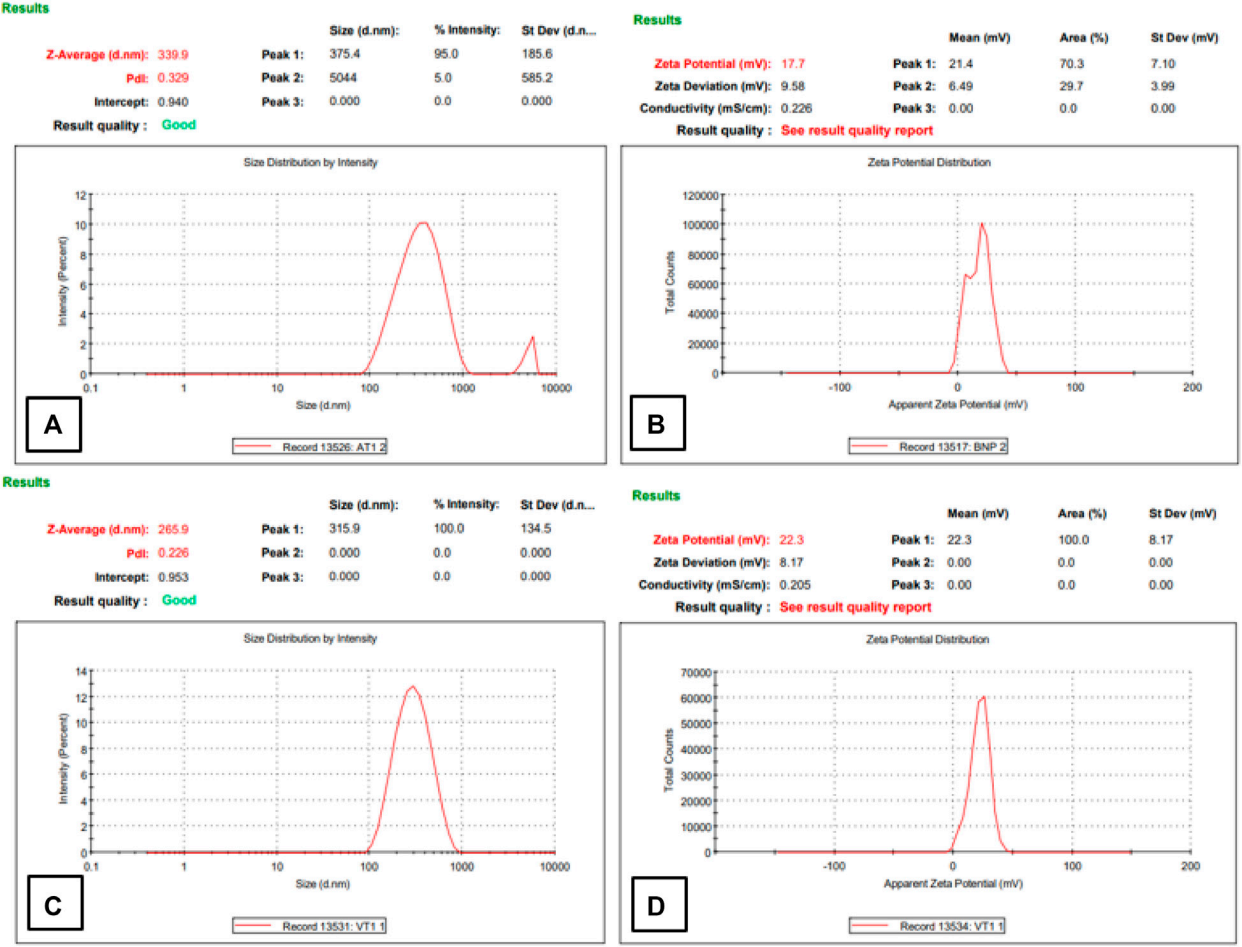
Particle size, PDI, and zeta potential of NFs of Blank **(A,B)** and Cis-loaded in HA-ThCs **(C,D)**.

Physiochemical properties like particle size, zeta potential, and PDI were analyzed by using a zeta sizer. Table 1 shows the analysis output of optimized HA-ThCs-Cis NPs. For blank HA-ThCs NPs, average particle size was 340nm, PDI .35, with zeta potential of 19 ± 1.05 mV at a concentration of .5 mg/mL of ThCs. For HA-ThCs-Cis NPs, the size and zeta potential of nanoparticles showed polymer concentration dependent decrease from .1 to 1 mg/ml formulations while the concentration of Cis was kept constant. The smallest observed particle size for HA-ThCs-Cis was 265.9 nm with PDI of .226 and zeta potential of 22.3 ± .81 mV at a concentration of .5 mg/ml of ThCs and .5 mg/ml of Cis as shown in Figure 6 and Table 1.

**TABLE 1:** Physiochemical characteristics of blank ThCs and Cis loaded ThCs nanoparticles.

Sr.	Formulations	Particle size (nm)	PDI (mV)	Zeta potential
01	Blank NF	340	.35	19 ± 1.05 mV
02	Cisp-loaded in ThCs-HA NF	265.9	.22	22.3 ± .81 mV

#### 3.4.2 Morphology of NPs formulation

**FIGURE 7:**
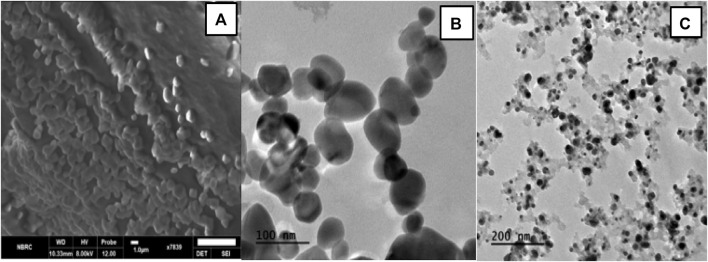
**(A)** SEM images show spherical HA-ThCs-Cis loaded nanoparticles at scale of 1um. **(B)** Spherical shaped and smooth HA-ThCS-Cis nanoparticles at scale of 100 nm. **(C)** Blank HA-ThCs NPs formulation at scale of 200 nm.

To analyze the surface and shape of the nanoparticles, TEM and SEM analysis of blank and Cis loaded nanoparticles was done. The results revealed that blank and Cis-loaded HA-ThCs nanoparticles have smooth surfaces. Both SEM and TEM images show that NPs have a spherical shape as shown in Figure 7.

#### 3.4.3 X-ray crystallographic analysis of NFs

**FIGURE 8:**
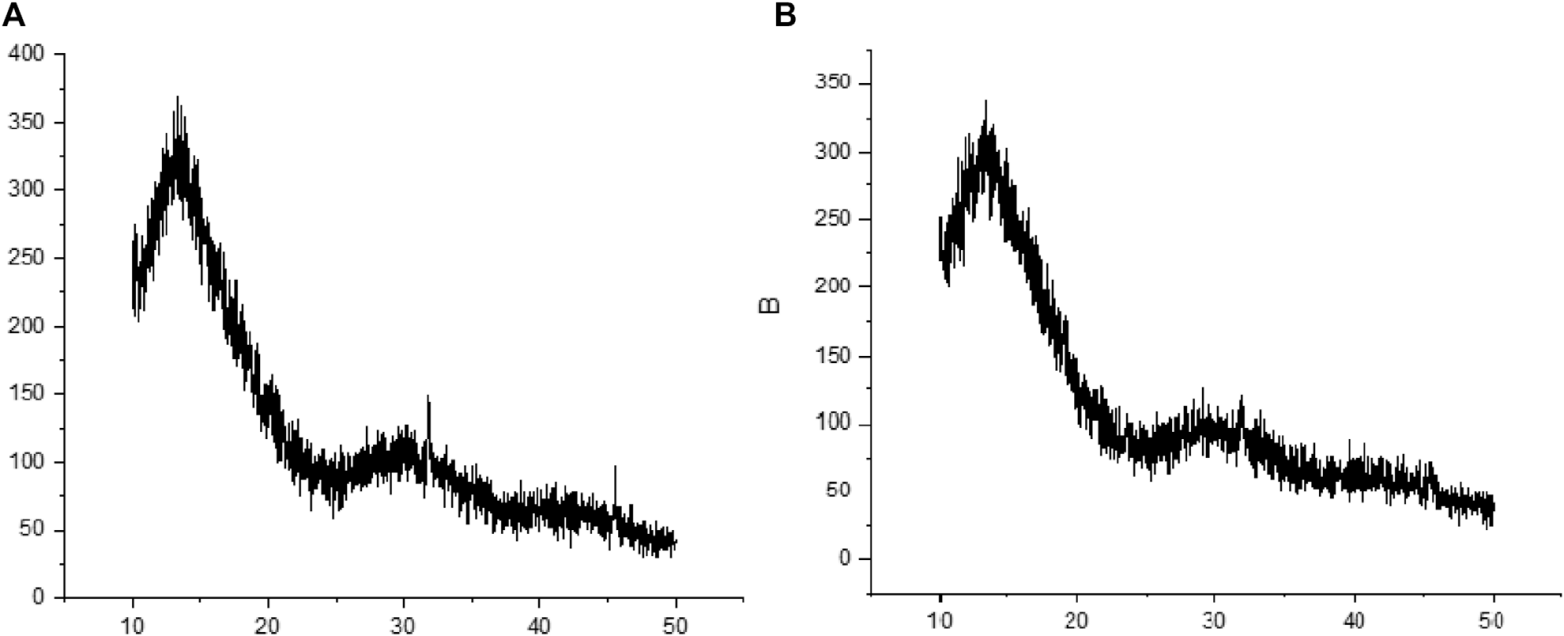
XRD study indicated crystalline nature of Blank HA-ThCs NPs **(A)** HA-ThCs-Cis NPs **(B)**.

XRD analysis was done to evaluate the impact of extra molecular and intermolecular interactions on crystalline blank and HA-ThCs-Cis NPs formulations. X-ray diffraction is used to explicate the crystallinity of the particles, it is a non-destructive method. The prominent reflection was seen at 2θ = 14.9° while a slight reflection was observed at 25° as depicted in Figure 8.

#### 3.4.4 FTIR for identification of functional group

**FIGURE 9:**
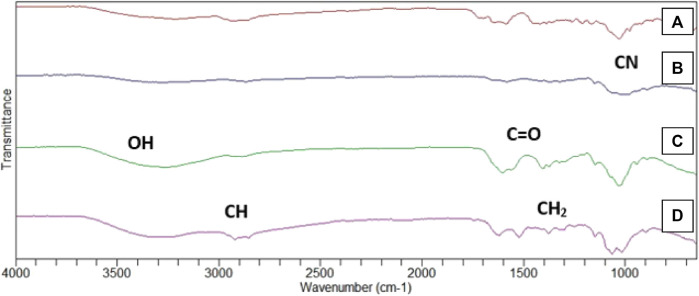
FTIR spectrum of Blank **(A)** HA-ThCs-Cis **(B)** HA **(C)** ThCs **(D)** nanoparticles.

The FTIR spectra gave information about the phase composition and presence of characteristics peaks in blank and Cis loaded HA-ThCs NPs formulation as shown in Figure 9. At 3,390 cm^−1^, stretching was observed due to the presence of the OH group, followed by another stretching at 2,900 cm^−1^ which indicated the presence of CH bond, the presence of C=O bond can be observed due to stretching in spectra at 1,650 cm^−1^, at 1,400 cm^−1^ due to the presence of CH_2_ group and then the presence of CN can be observed due to stretching between 1,050 and 1,100 cm^−1^. The slight fluctuation around 2,500 cm^−1^ indicates the presence of the thiol group.

#### 3.4.5 Raman spectroscopic analysis

**FIGURE 10:**
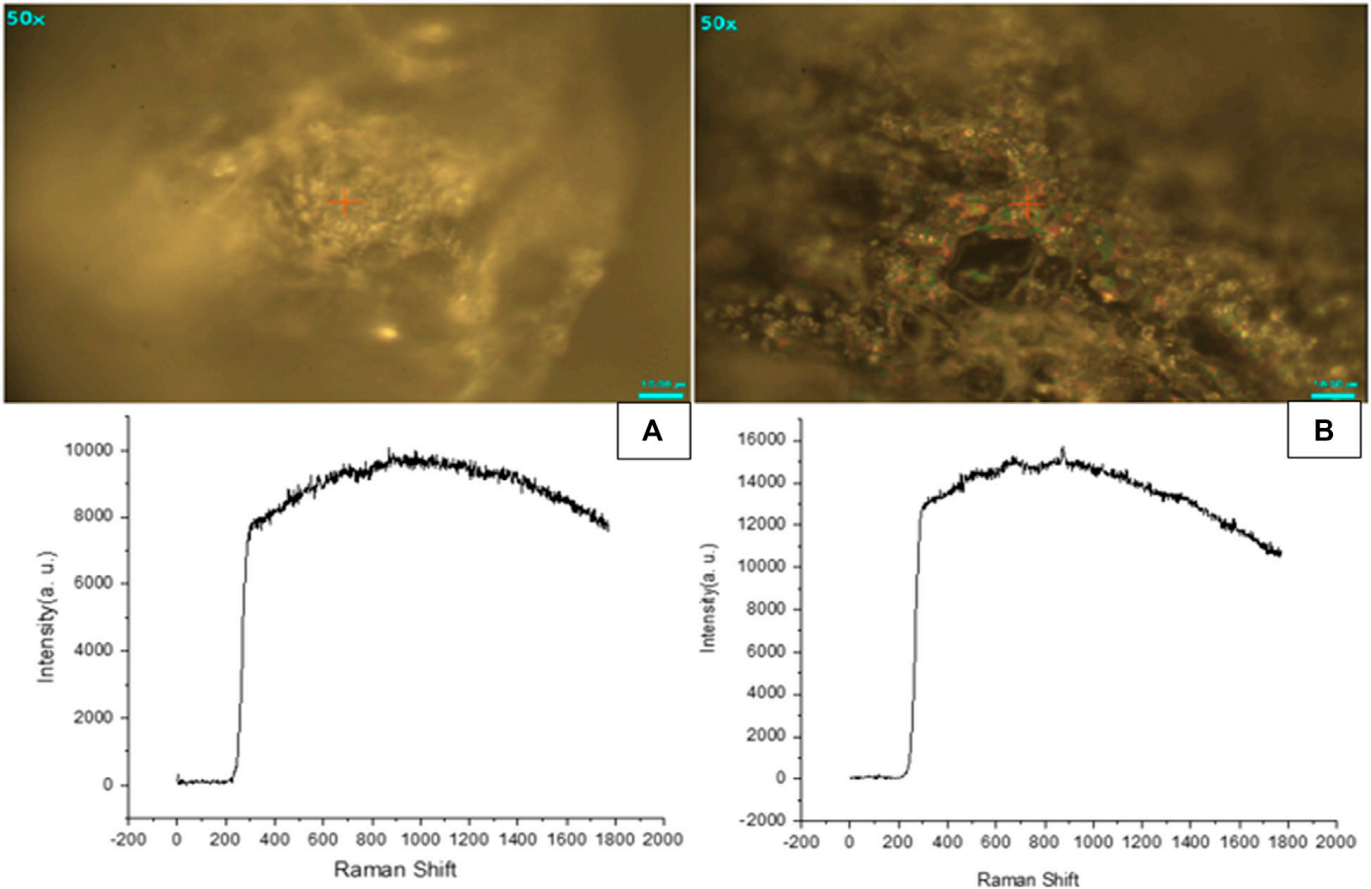
Raman spectroscopic analysis showed that Blank NFs **(A)** and HA-ThCs-Cis NFs **(B)** has porous surface.

Raman analysis gives insight into the vibrational modes of molecules. As shown in Figure 10, main peaks were observed at 330 cm^−1^ due to the bending vibration of C-C-O. At around 910 cm^−1^ a very slight deflection was observed, after 1,000 cm^−1^. The deflection pattern begins to vary more in blank and Cis loaded HA-ThCs NPs formulation. The deflection pattern of Cis-loaded NPs formulation is similar to what was observed in the FTIR spectrum of blank and Cis-loaded NPs formulation. The deflection pattern of HA-ThCs NPs formulation indicates that agglomeration features provided these nanoparticles a porous surface making them suitable as nano drug carriers.

#### 3.4.6 Drug loading and encapsulation efficiency

UV- visible spectroscopy was used to quantify the amount of Cis loaded in HA-ThCs nanoparticles. The average values of the percentage of drug loading (DL) and encapsulation efficiency (EE) for Cis loaded in HA-ThCs were 70.1% ± 1.2% and 45% ± .28% respectively, as shown in Table 2 below.

**TABLE 2:** Percentages of EE and DL of NFs of Cis loaded in HA-ThCs (*p* < .05 mean ± S.D).

Absorbance	Concentration (mg/mL)	EE (%)	EE% (mean ± SD)	DL	DL% (mean ± SD)
1.244	.006	68.74	70.1 ± 1.2	.49	45 ± .28
1.566	.008	71.72		.43	
1.973	.01	69.84		.45	

#### 3.4.7 *In vitro* drug release and kinetic model

**FIGURE 11:**
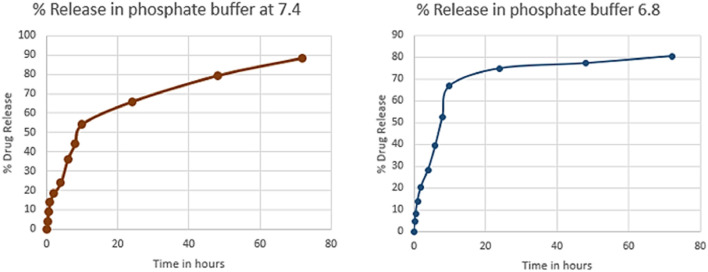
% drug release of Cis from HA coated ThCs nanoparticles at pH 6.8 and 7.4.

**FIGURE 12:**
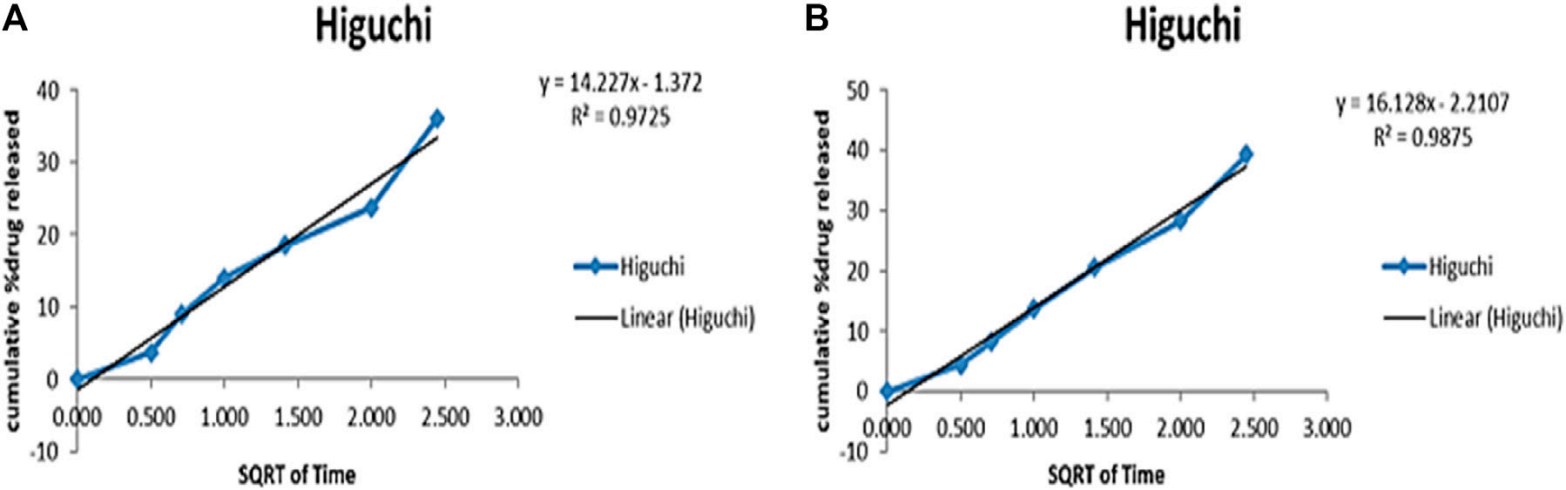
The best release model Higuchi graph for Cis release from HA-ThCs-Cis NFs at pH 6.8 **(A)** and 7.4 **(B)**.

To evaluate the release profile of Cis from NPs at pH 6.8 (tumor microenvironment) and 7.4 (normal physiological pH), a release study was conducted and compared with normal Cis release in buffer at 7.4 pH. For time intervals of 10, 24, 48 and 72 h, the percentage of drug released from NPs formulation at pH 6.8 was 66.8%, 74.8%, 77.2% and 88% respectively as compared to 54.2%, 65.9%, 79.3% and 80.5% respectively at pH 7.4 (Supplementary Table S3). Pure drug was released within 8 h while this release profile shows sustained release of drug from nanoparticles for upto 72 h. The release of Cis is shown in Supplementary Table S3 at pH 6.8 and 7.5 at different time intervals. Figure 11 shows release pattern of Cisplatin from nanoformulation at both pH. For elaborating the mechanism of drug release from NPs formulation at both pH 6.8 and 7.4, different kinetic models were applied as shown in Supplementary Table S4. Higuchi square root of time equation came out to be the best drug release model as compared to other kinetic models because of R^2^ value very close to 1 (Figure 12). Higuchi model of our HA-ThCs-Cis NFs explains the release of drug by simple diffusion mechanism, with no dissolution of matrix during process and constant diffusivity of the drug.

### 3.5 Cell viability analysis of NFs

**FIGURE 13:**
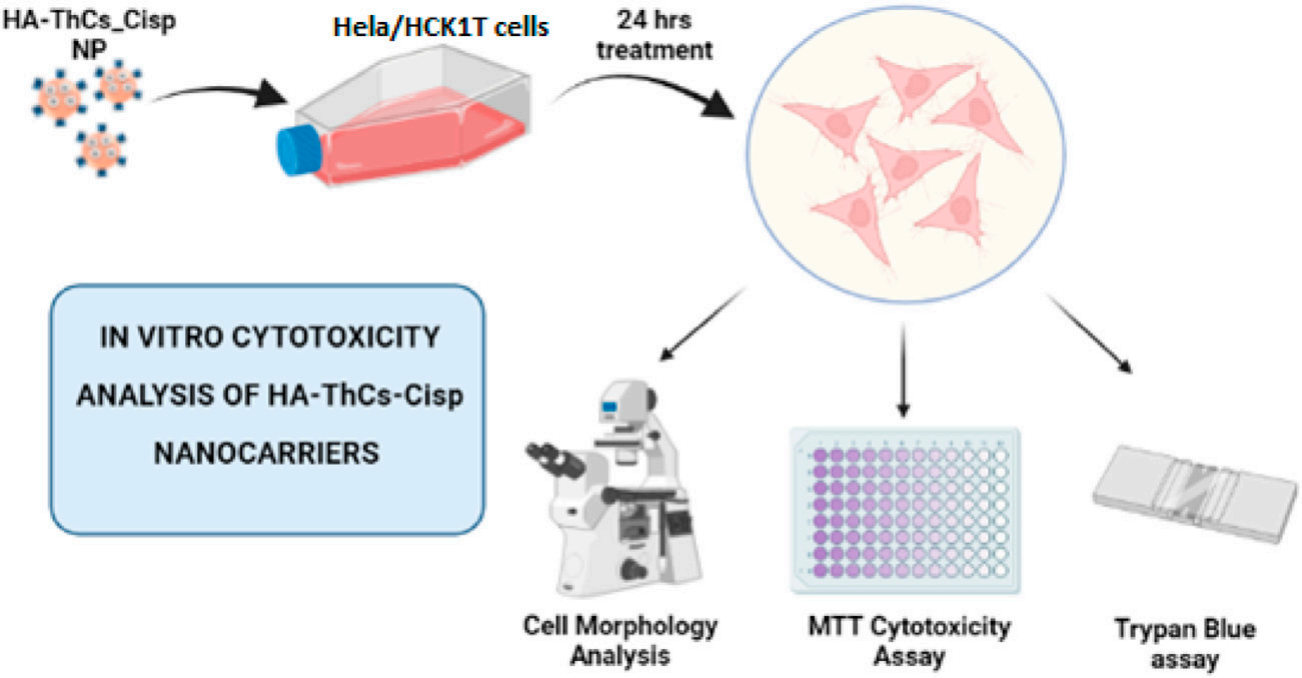
*In vitro* analysis of cytotoxic potential of HA-ThCs-Cis nanoparticles on normal cervical (HCK1T) and cervical cancer (Hela) cell lines.

Cell viability analysis was carried out at different dose concentrations of 10, 40 and 80 μg/ml for pure Cis and HA-ThCs-Cis nanoparticles on cervical cancer HeLa cells and the results were compared with normal cervical cells HCK1T cells treated with same dose. For this purpose, cell morphology analysis, trypan blue exclusion assay, and MTT assay were performed (Figure 13).

#### 3.5.1 Cell morphology analysis

**FIGURE 14:**
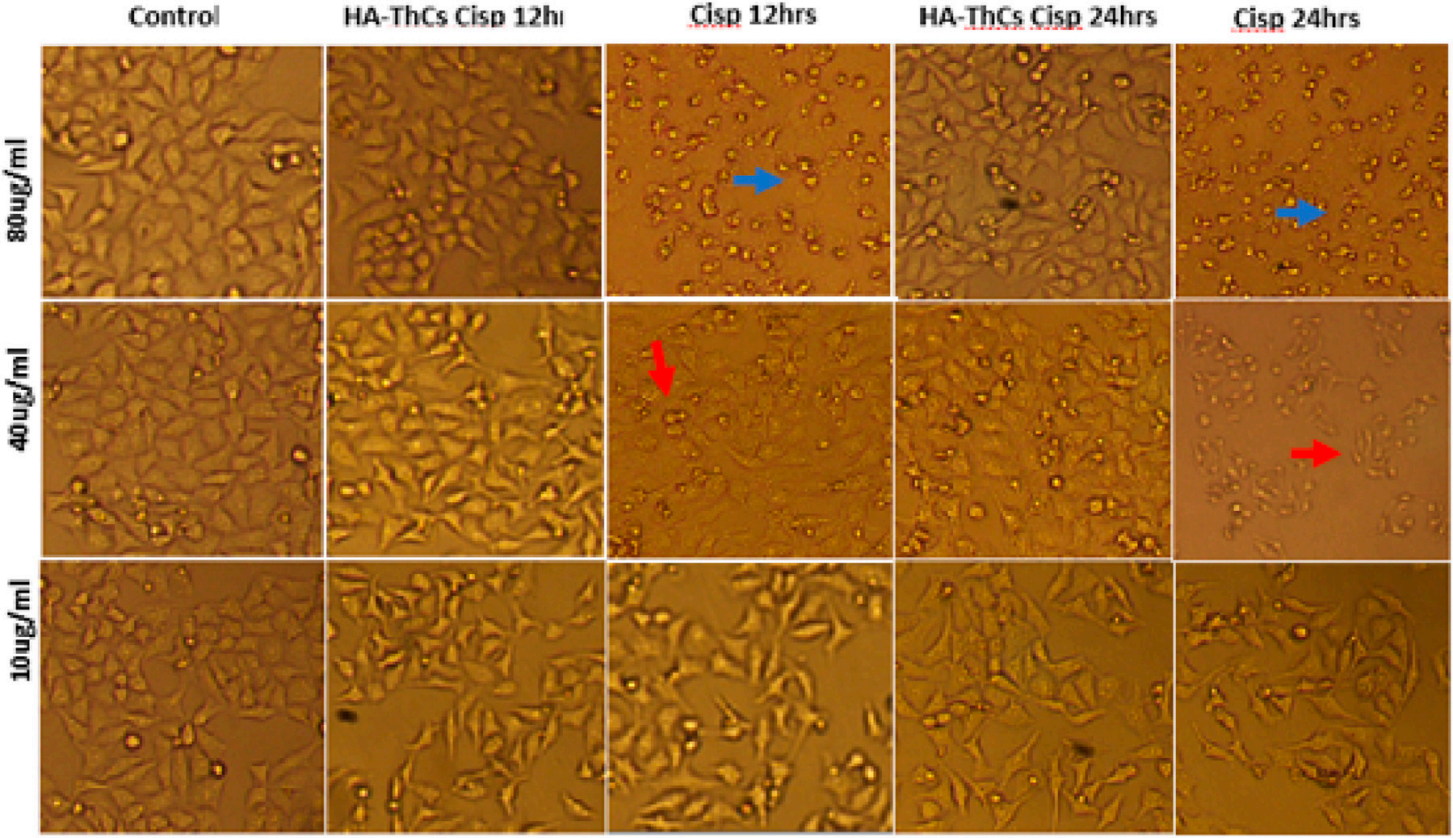
Morphological changes under inverted microscope (10x) in HeLa cells treated with HA-ThCs-Cis and pure Cis at time intervals of 12 and 24 h. Blue arrowheads show rounding and aggregation, while red arrowheads show cell shrinkage.

**FIGURE 15:**
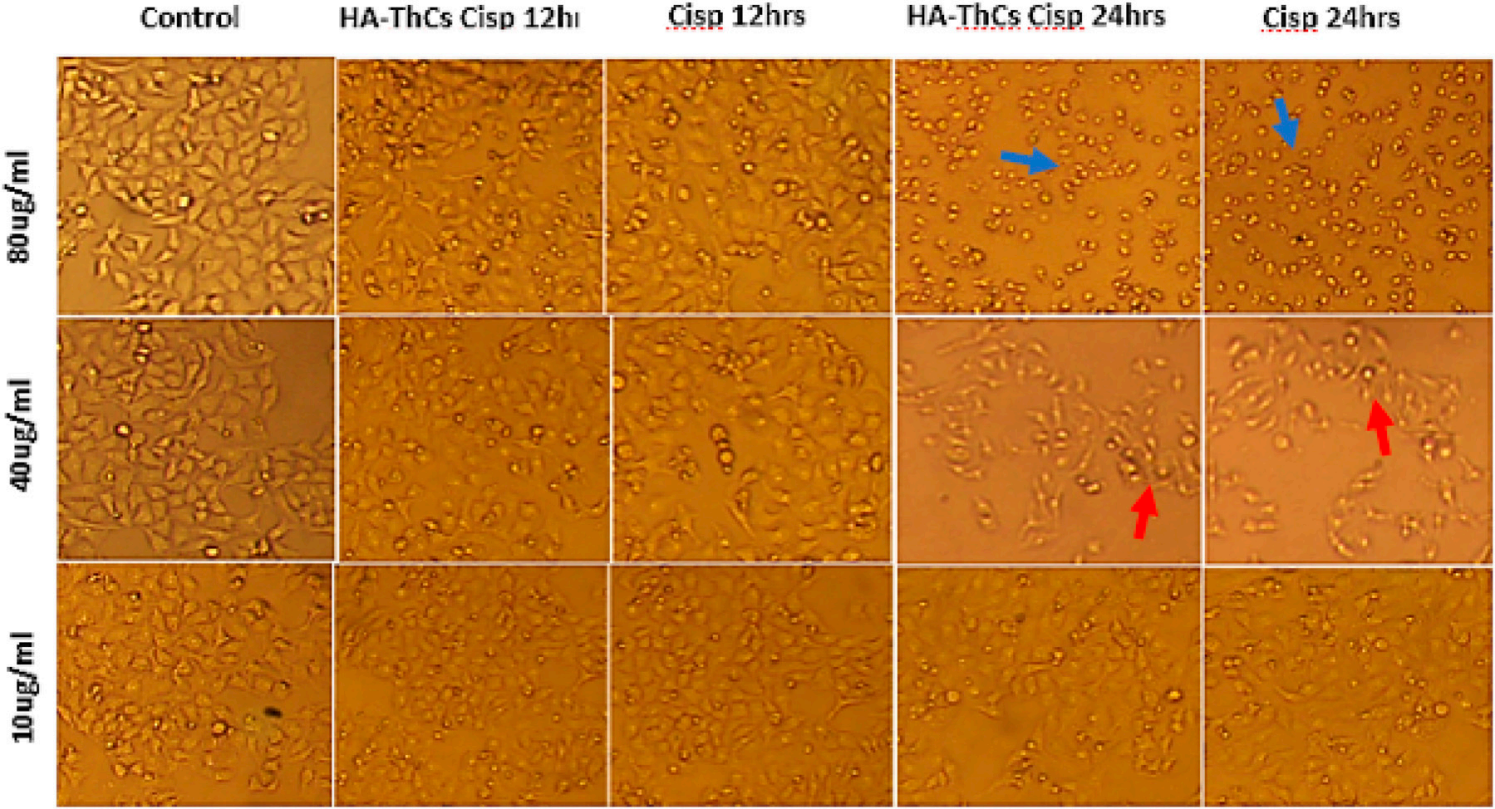
Morphological changes under inverted microscope (10x) in HCK1T cells treated with HA-ThCs-Cis and pure Cis at time intervals of 12 and 24 h. Blue arrowheads show rounding and aggregation, while red arrowheads show cell shrinkage.

HeLa cells when treated with different concentrations of HA-ThCs-Cis, blank NPs, and Cis, exhibited a clear change in morphology and underwent apoptosis. HeLa Cells treated with HA-ThCs-Cis showed cytotoxic effect in a dose and time-dependent manner. As shown in Figure 14, control cells can be compared with treated HeLa cells at time intervals of 12 and 24 h to observe a change in cell morphology and detachment after treatment with HA-ThCs-Cis and pure Cis. The highest apoptotic effect was observed at the time interval of 24 h when treated with 80 μg/mL concentration. On other hand, HCK1T cells when treated with Cisplatin exhibited high cytotoxic effect which increased in dose dependant manner, while the cytotoxic effect of ThCs-Cis nanoparticles on HCK1T cells at same concentration were very less comparatively (Figure 15). For Hela cells, the IC50 for pure Cis was calculated to be 8 μg/ml for 12 h and 19 μg/ml for 24 h treatment. While the IC50 for HA-ThCs-Cis nanoformulation was calculated to be 6 μg/ml for 12 h and 13 μg/ml for 24 h treatment. For HCK1T, the IC50 for pure Cis was calculated to be 10 μg/ml for 24 h and 23 μg/ml for 48 h treatment. While the IC50 for HA-ThCs-Cis nanoformulation was calculated to be 18 μg/ml for 12 h and 35 μg/ml for 24 h treatment. In Figures 14, 15, blue arrow heads indicate rounding and aggregation, while red arrow heads indicate shrinkage of cells.

#### 3.5.2 Trypan blue exclusion assay

Trypan blue exclusion assay was performed to assess apoptosis in cells treated with Cis-loaded NF and pure Cis at different concentrations. As shown in Supplementary Table S5, % cell viability in HeLa cells increased with decrease in concentration of NF. Maximum % cell viability of 85% ± .03% was observed at a concentration of 10 μg/ml and minimum cell viability of 12% ± .05% was observed at a concentration of 80 μg/ml for HA-ThCs-Cis treatment. On other hand, Hela cells showed maximum % cell viability at concentration of 90 ± .12 at 10 μg/ml and minimum % cell viability 15 ± .81 at concentration of 80 μg/ml when treated with pure Cisplatin. For HCK1T cells treated with HA-ThCs-Cis, highest %cell viability of 83 ± .3 was observed at concentration of 10 μg/ml, while lowest %cell viability of 59 ± 1.4 was observed at concentration of 80 μg/ml. In pure Cisplatin treated HCK1T cells, Highest %cell viability of 78 ± .1 was observed at concentration of 10 μg/ml, while lowest %cell viability of 15 ± .6 was observed at concentration of 80 μg/ml. All experiments were performed in triplicates.

#### 3.5.3 MTT Cytotoxicity assay

**FIGURE 16:**
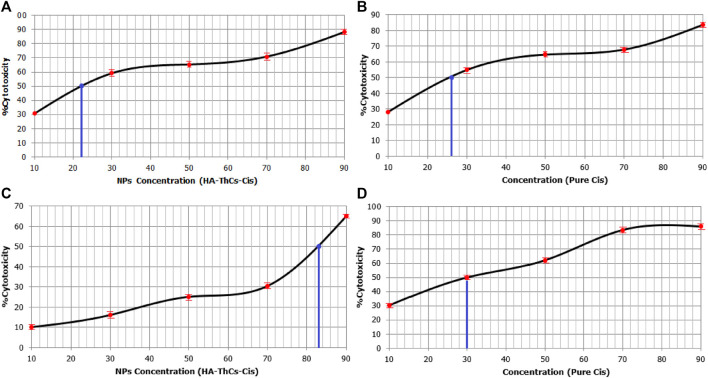
Sigmoid curves show MTT results for % cytotoxicity of HeLa cells treated with HA-ThCs-Cis **(A)** Pure Cis **(B)** and HCK1T cells treated with HA-ThCs-Cis **(C)** and Pure Cis **(D)**. Blue line indicates IC50 value. X-axis shows concentration of NPs and Cis in µg/mL and Y-axis shows % cytotoxicity.

Cell viability of NF and Cis-treated cells was analyzed through MTT cell viability analysis. Cells were treated with different concentrations of 10, 30, 50, 70, and 90 μg/ml of pure Cis and NPs formulation. For HeLa cells, 90 μg/ml concentration showed the highest cellular toxicity of 83.71% ± .03% for pure Cis and 88.21% ± .04% for NFs, while minimum cytotoxicity was observed at a concentration of 10 μg/ml, where pure Cis showed 28% ± .07% cytotoxicity while HA-ThCs-Cis showed 30.72% ± .01% cytotoxicity as shown in Table 1S (Supplementary Material) and Figures 16A, B. For HCK1 T cells, 90 μg/ml concentration showed the highest cellular toxicity of 85.9% ± .8% for pure Cis and 25% ± .03% for NFs, while minimum cytotoxicity was observed at a concentration of 10 μg/ml, where pure Cis showed 30.3% ± .6% cytotoxicity while HA-ThCs-Cis showed 10% ± .2% cytotoxicity as shown in Table 1S (Supplementary Material) and Figures 16C, D. In Figure 16, blue line indicates IC50 of HA-ThCs-Cis and Pure Cis.

### 3.6 Nanoparticle stability analysis

**FIGURE 17:**
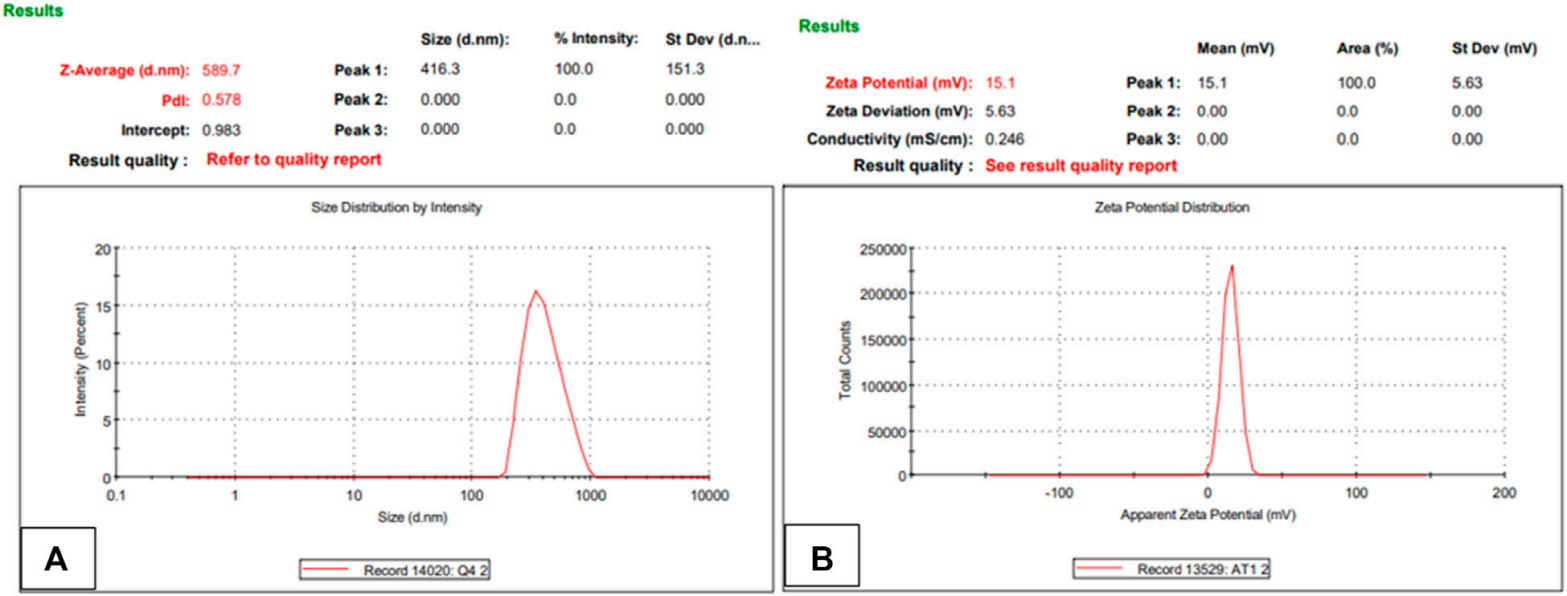
Nanoparticle size, PDI **(A)** and zeta potential **(B)** of NPs formulation after redistributing lyophilized powder in deionized water (*n* = 3).

The stability of the nanoparticles in terms of particle size, PDI, zeta potential and shape were analyzed after 3 months while NPs were stored at temperatures of 37°C and 4°C. It was observed that lyophilized powder did not undergo any change in surface characteristics while nanoparticles in the liquid formulation had changed in particle size from 265.9 to 589.7 with PDI change from .2 to .57 and zeta potential from +22.3 to +15.1 mV as shown in Figure 16 below (Figures 16A, B). The drug loading and encapsulation efficiency also changed from 70.1% ± 1.2% to 64.51% ± .32% and from 45% ± .28% to 40% ± .47% respectively. All experiments were performed in triplicates. These findings suggest that this nanocarrier is suitable for long-term storage in lyophilized powder state (Figure 17).

## 4 Discussion

Molecular docking studies to assess binding interaction between hyaluronic acid and CD44 depicted a strong and viable binding. The binding energy of hyaluronic acid with 4PZ3 (−7.2 kcal. mol) shows stronger binding with favourable number (five) of hydrogen bonds (Naseer et al., 2021).

Ellman’s reagent of DTNB is very sensitive to the reaction of thiol sulfide exchange with the free thiol group, shows high specificity at neutral pH and helps in quantitative measurement of free sulfhydryl groups, therefore it is also called sulfhydryl assay. Our one step thiolation reaction yielded 787 µmol of thiol groups present per Gram of polymer. These results are in line with findings of who reported that thiolation of chitosan yielded 465 μmol/G of polymer (Shahid et al., 2022).

Nanoparticles were prepared through ionotropic gelation method and this method produced spherical shaped, stable, drug loaded nanoparticle which were later characterized for physiochemical properties and therapeutic efficacy in cancer cells. In a previous study, it was reported that HA-coated chitosan nanoparticles carrying tamoxifen were prepared through the ionic gelation method for targeted delivery in MCF-7 and tamoxifen-resistant MCF-7 cell lines *in vitro* (Nokhodi et al., 2022).

Particle size, shape, and surface chemistry greatly influence onsite delivery, stability, toxicity, biodistribution, cellular uptake, the pharmacokinetics of the drug, and even the *in vivo* performance of nanoparticle-mediated DDS (Jindal, 2017). The nanoparticles showed spherical morphology which is considered ideal in nano based drug delivery systems. Nanoparticle size of 265.2 nm shows that HA-ThCs-Cis nanoparticles have favorable size to accumulate in tumor microenvironment through enhanced permeation and retention effect (EPR). EPR is an important driver in cancer nanomedicine and favours nanoparticles ranging between 50 nm and 500 to squeeze through leaky vessels of tumor vasculature and accumulate at the tumor site (Chen & Cai, 2014). Also, the reticuloendothelial system which consists of phagocytic cells shows a low rate of clearance of particles with size below 300 nm, this enhances the circulation time of these nanoparticles leading to enhanced uptake by tumor cells (Baranov et al., 2021). Our results are in conformity with the study in which spherical-shaped, 250 nm-sized curcumin-loaded poly (butyl) cyanoacrylate (PBCA) nanoparticles showed effective anticancer activity against Vero cells (Deepika et al., 2022). According to another published data, spherical-shaped nanoparticles are stable in blood and flow conveniently with blood whereas non-spherical nanoparticles flip as the blood flows (Eliezar et al., 2015).

The zeta potential of 22.3+ mV on nanoparticles will favour their uptake by the negative charge on the cellular membrane of tumor cells. The PDI of our selected nanoformulation was .226 which shows high uniformity among particles of nanoformulation. Polydispersity index (PDI) determines the homogeneity of particles in NPs formulation and a value below .5 exhibits uniform size distribution of nanoparticles while a PDI value above .5 shows heterogeneity in the particle size (Danaei et al., 2018). Curcumin loaded mucoadhesive ThCs nanoparticles with a diameter between 200 ± 50 nm and zeta potential of 11–38 mv were reported for sustained release of curcumin in VFS (vaginal fluid simulant), this resulted in sustained release of curcumin for up to 3 days depicting enhanced permeation, retention, less clearance and mucoadhesive nature of ThCs nanoparticle DDS (Hasanifard et al., 2017). In our study, similar sustained release of drug was recorded and a release profile of the Cis from <300 nm HA-ThCs-Cis NPs till 72 h (Figure 11) indicates the NPs formulation favours sustained release consequently increasing the bioavailability of the drug and favours a decrease in drug dosage, ultimately reducing dose-related side effects of the drug.

XRD exhibits crystalline structure, grain size, and phase nature of crystalline/semi-crystalline material. These diffraction patterns are used to build a quality image of the topology of atoms within the crystal lattice of the material under study (Mukhtar et al., 2020). The similar XRD pattern of blank and Cis-loaded HA-ThCs nanoparticles indicates that intermolecular interactions, hydrogen bonding, and extra molecular interactions were retained during the process of loading in NPS formulation. The diffraction peak of chitosan are in line with findings of a reported study in which 5-Fluorouracil and curcumin-loaded chitosan/reduced graphene oxide nanoparticles showed major diffraction at 20.04° (Dhanavel et al., 2019).

FTIR analysis showed presence of characteristic peaks and further confirmed the successful encapsulation of Cis inside the nanoparticles. This FTIR result is in accordance with reported study in which the FTIR spectrum of curcumin-loaded ThCs nanoparticles showed similar peaks as observed in our FTIR analysis.

Raman spectroscopic analysis is a non-destructive method and provides a structural fingerprint for the identification of molecules (Ren et al., 2014). Raman analysis also confirmed the presence of characteristic functional groups and showed that our nanoparticles are porous in nature. According to previous data, peaks at 393 cm^−1^ showed C-C (=O)-C, at 596 cm^−1^ showed C-C=O, at 839 cm^−1^ showed C-O-C, at 969 cm^−1^ showed CH group, at 1,148 cm^−1^ showed C-N, at 1,454 cm^−1^ showed CH_3_ and at 1,550 cm^−1^ showed NH_2_ functional groups (Ren et al., 2014). Porous nanoparticles are known to exhibit high loading capacity, better tumor tissue targeting, tuneable porosity, low immune clearance, and immune-related adverse events making them promising nano drug delivery system (Li et al., 2022).

HA-ThCs-Cis nanoparticles exhibited good drug loading and encapsulation efficiency and their biocompatible nature makes them an ideal non-toxic nanocarrier to deliver drugs into cancer cells. As reported, the high encapsulation efficiency of the drug in nanoparticles increases bioavailability and gives a better therapeutic outcome with the least side effects and a beneficial overall pharmacokinetic profile (El-Say, 2016). According to another study, chitosan was used to encapsulate Cis with a final encapsulation efficiency of 83.3% ± 1.5% (Sultan et al., 2022). In our case, we achieved 70.1% **±** 1.2% EE which indicate an effectual encapsulation of the Cis. In another report, the EE and DL efficiency of chitosan nanoparticles encapsulating 5-fluorouracil was 44.28% ± 1.69% and 20.13% ± .007% respectively when chitosan and 5-Fluorouracil was used in the mass ratio of 1:1 (Xu et al., 2013).

Our nanoparticle formulation showed sustained release for up to 72 h. It has been reported that improved effect of NPs-loaded doxorubicin was observed as compared to free drug leading to enhanced cell cycle arrest and apoptosis (Nogueira-Librelotto et al., 2020). These findings are also supported by the results of another study which reported that DOX-TTP loaded polymeric polylactic-co-glycolic acid (PLGA) at a mildly acidic environment of 6.8 provided sustained release over a longer period of time (Palanikumar et al., 2020). Higuchi model of drug release shows that nanoformulation release drug through diffusion. The release of drug for longer time intervals show stable NPs formulation and sustained release of Cis (Pourtalebi Jahromi et al., 2020).

*In vitro* analysis of cancer cells HeLa and normal cervical epithelial cells HCK1T treated with HA-ThCs-Cis nanoparticles showed cytotoxic effect in time and dose dependent manner. Cellular morphology is the first indicator of the healthy state of cells. When cells are treated with cytotoxic drugs they shrink, their nuclei shrink, the membrane becomes disintegrated, and they lose their characteristic morphology exhibiting the hallmarks of apoptosis. A shown in Figure 14 and Figure 15 respectively, HeLa cells exhibited hallmarks of apoptosis when treated with HA-ThCs-Cis while HCK1T cells, treated with same dose of nanoformulation showed comparatively normal morphology. Our findings of cell morphology analysis are in coherence with the findings of who reported that Cis-induced cellular distortion in a dose-dependent manner when used alone and in combination with ethanol extract of *Peumus boldus* against liver cancer *in vitro* and *in vivo* (Mondal et al., 2014). The findings of trypan blue exclusion assay which shows that HeLa cells exhibited low cell viability when treated with HA-ThCs-Cis while HCK1T showed high cell viability are also in line with findings of who reported that that polymorphic nuclear cells (PMN) treated with cis-dichloroplatinum (II) complex [(Qu)2PtCl2 dose-dependently induce membrane degradation, with the highest cell viability of 77% observed at a concentration of 17 μg/mL (Hussein and Mohsin Jasim, 2019). In our study, the highest viability was measured at 10 μg/ml and the lowest at 80 μg/mL. MTT cytotoxicity assay further confirmed the findings of cell morphology analysis and trypan blue exclusion assay in which Hela cells showed highly cytotoxic behaviour while cytotoxicity in HCK1T cells was very low for HA-ThCs-Cis treated cells. After 24 h treatment, IC50 for HeLa treated with HA-ThCs-Cis was 22 μg/ml, while for pure Cis IC50 was 26 μg/ml. For HCK1T treated with HA-ThCs-Cis, IC 50 was calculated to be 83 μg/ml while IC50 for pure cis treated HCK1T cells was 30 μg/ml. Greater cytotoxicity in HeLa, as compared to HCK1T for NPs treatment explains the enhanced uptake of receptor-mediated drug-loaded nanoparticles A similar study was performed using Cisplatin loaded chitosan nanoparticles and Cisplatin loaded rituximab surface linked nanoparticles for targeted delivery of Cisplatin inside breast cancer cells MCF-7. Our finding is in coherence with their findings as their nanoformulation showed sustained release, better *in vitro* cytotoxicity on MCF-7 cells but due to antibody surface functionalization, targeting capacity could not be validated for breast cancer cells (Sultan et al., 2022). As reported in another study, Catechol modified chitosan nanoparticles, surface functionalized with hyaluronic acid were used for targeted delivery of Doxorubicin in oral cancer. While catechol and chitosan supported adhesion of nanoparticles to oral mucosa, surface functionalization with HA decreased off-target toxicities of DOX. These 160 nm sized nanoparticles had high loading capacity with ability of sustained release and enhanced cellular uptake. Their findings also suggest the efficacy of HA functionalized, Cs nanoparticles in targeted delivery, sustained release and enhanced cellular uptake by cancer cells (Pornpitchanarong et al., 2020).

These findings prove that CD44 targeted, thiolated chitosan drug delivery system in cancer can lead to reduced off-target toxicity events with sustained release for longer period of times, ultimately yielding a better therapeutic potential of the anticancer drug Cis. Stability test conducted after 3 months further depicted that nanoformulation used in the study is stable in lyophilized form for storage.

## 5 Conclusion

Nanoparticle-mediated drug delivery system has completely changed the dynamics of targeted ThCs have proved to be a biocompatible, mucoadhesive polymer that can encapsulate different treatment modalities like drugs, antibodies, and oncolytic viruses for cancer. CD44 targeting through HA-mediated surface functionalization turned out to be an additional beneficial characteristic of HA-ThCs nanoparticles used in this research. Both biopolymers are non-toxic, non-immunogenic, biocompatible, biodegradable and therefore do not pose harm in terms of undesired side effects. The particle size of our nanoparticle below 300 nm (265.9 nm) with a positive net charge is particularly useful for targeted drug delivery and retention at the tumor site through enhanced permeation and retention effect and due to dominant negative charge on the membrane of the cancer cells. The drug-loaded NPs formulation exhibited good encapsulation efficiency and a favourable stability profile. *In vitro* analysis of NPs formulation in the HeLa cervical cancer cell line and showed better cytotoxicity as compared to normal HCK1T cells in a time and dose dependant manner as compared to conventionally used pure Cis (*p* < .05), and thus proves a proficient CD44 mediated nanoparticle uptake in the cancer cells. Analysis of the drug release profile at pH 6.8 and 7.4 showed that drug release follows Higuchi model with sustained release for upto 72 h. All these findings prove that HA-ThCs-Cis NPs formulation is a promising drug delivery system for active targeting of cancer cells through the CD44 receptor. As a future perspective, *in vivo* evaluation of the NPs formulation is strongly recommended as the next step towards an improved drug delivery system for cervical cancer.

## Data Availability

The original contributions presented in the study are included in the article/Supplementary Material, further inquiries can be directed to the corresponding authors.
