# Supplementary material for: Green synthesis of hyaluronic acid coated, thiolated chitosan nanoparticles for CD44 targeted delivery and sustained release of Cisplatin in cervical carcinoma
Source: Front Pharmacol. 2023 Jan 12;13:1073004. doi: 10.3389/fphar.2022.1073004 (PMC9877355; doi:10.3389/fphar.2022.1073004)
Supplement: Supplementary file 1 [file Table1.docx]

| **Name of protein (receptor)** | **PDB ID** | **Ligand** | **Binding energy ΔG (kcal/mol)** | **Amino acid residues** | **Distances**  **A°** | **Type of interaction** |
| --- | --- | --- | --- | --- | --- | --- |
| Human CD44 | 4PZ3 | Hyaluronic Acid | -7.2 | TYR A:79 | 3.34 | Conventional hydrogen bond |
|  |  |  |  | THR A: 108 | 3.44, 2.99 | Conventional hydrogen bond |
|  |  |  |  | TYR A: 105 | 3.03, 2.84 | Conventional hydrogen bond |
|  |  |  |  | LEU A: 107 | 3.29 | Van der Waals |
|  |  |  |  | SER A: 112 | 3.05, 4.52 | Conventional hydrogen bond |
|  |  |  |  | TYR A: 42 | 2.21 | Conventional hydrogen bond |
|  |  |  |  | GLN A: 113 | - | Van der Waals |
|  |  |  |  | THR A: 111 | - | Van der Waals |
|  |  |  |  | SER A: 109 | 2.80, 2.75 | Unfavourable acceptor-acceptor |

*Table 1S:Shows the binding energies, amino acid residues, binding distances and types of interaction among ligand and protein molecules*
