# Supplementary material for: Green synthesis of hyaluronic acid coated, thiolated chitosan nanoparticles for CD44 targeted delivery and sustained release of Cisplatin in cervical carcinoma
Source: Front Pharmacol. 2023 Jan 12;13:1073004. doi: 10.3389/fphar.2022.1073004 (PMC9877355; doi:10.3389/fphar.2022.1073004)
Supplement: Supplementary file 2 [file Table2.docx]

| Formulation number | HA mg | TCS mg | Cis mg | size | PDI | Zeta(mV) |
| --- | --- | --- | --- | --- | --- | --- |
| 1 | 5.00 | 1.00 | 1 | 806 | 0.681 | 5.65 |
| 2 | 2.75 | 10.00 | 0.1 | 680.4 | 0.416 | 4.88 |
| 3 | 5.00 | 5.50 | 0.5 | 339.9 | 0.329 | 15.1 |
| 4 | 0.50 | 1.00 | 0.5 | 265.9 | 0.226 | 22.3 |
| 5 | 0.50 | 5.50 | 1 | 736.1 | 0.528 | 13.3 |
| 6 | 0.50 | 5.50 | 0.1 | 614 | 0.401 | 13.8 |
| 7 | 5.00 | 5.50 | 0.1 | 463.9 | 0.363 | 16.4 |
| 8 | 0.50 | 10.00 | 1 | 648.8 | 0.662 | 13 |
| 9 | 2.75 | 5.50 | 0.5 | 490.2 | 0.252 | 17.3 |
| 10 | 5.00 | 10.00 | 0.5 | 513.4 | 0.289 | 17.5 |
| 11 | 2.75 | 5.50 | 0.5 | 339.4 | 0.363 | 18.1 |
| 12 | 2.75 | 10.00 | 0.1 | 629.4 | 0.394 | 15 |
| 13 | 2.75 | 5.50 | 1 | 376.2 | 0.455 | 19.2 |
| 14 | 2.75 | 1.00 | 0.1 | 371.2 | 0.322 | 17.7 |
| 15 | 2.75 | 1.00 | 0.5 | 267.9 | 0.21 | 17.8 |

*Table S2: DOE run parameters for optimization of nanoformulation that included different HA and ThCs concentrations with variable particle size, Sonication time, zeta potential and PDI. The selected values show the best optimized formulation.*
