# Supplementary material for: Green synthesis of hyaluronic acid coated, thiolated chitosan nanoparticles for CD44 targeted delivery and sustained release of Cisplatin in cervical carcinoma
Source: Front Pharmacol. 2023 Jan 12;13:1073004. doi: 10.3389/fphar.2022.1073004 (PMC9877355; doi:10.3389/fphar.2022.1073004)
Supplement: Supplementary file 3 [file Table5.docx]

| **S.No** | **Concentration** | **HA-ThCs-Cisplatin** | **Cisplatin** |
| --- | --- | --- | --- |
|  |  | **% Cell Viability Hela** | |
| 1 | 80 µg/mL | 12±0.05 | 15±0.81 |
| 2 | 40 µg/mL | 43±0.81 | 45±0.63 |
| 3 | 10 µg/mL | 85±0.03 | 90±0.12 |
| 4 | Control | 93±0.07 | 93±0.07 |
| **% Cell Viability HCK1T** | | | |
| 5 | 80 µg/mL | 59±1.4 | 15±0.6 |
| 6 | 40 µg/mL | 74±1.9 | 32±0.9 |
| 7 | 10 µg/mL | 83±0.3 | 78±0.1 |
| 8 | Control | 89±0.09 | 89±0.09 |

*Table 7: Trypan blue exclusion result of % cell viability of Hela and HCK1T (p<0.05, mean + S.D, n=3)*
