# Supplementary material for: Green synthesis of hyaluronic acid coated, thiolated chitosan nanoparticles for CD44 targeted delivery and sustained release of Cisplatin in cervical carcinoma
Source: Front Pharmacol. 2023 Jan 12;13:1073004. doi: 10.3389/fphar.2022.1073004 (PMC9877355; doi:10.3389/fphar.2022.1073004)
Supplement: Supplementary file 4 [file Table3.docx]

| **Sr. no.** | **Time**  **(Hr.)** | **cumulative % drug released at pH 7.4** | **cumulative % drug released at pH 6.8** |
| --- | --- | --- | --- |
| 1 | 0 | \| 0 \| \| --- \| | 0 |
| 2 | 0.25 | 4.485±0.5 | 3.728±1.2 |
| 3 | 0.5 | \| 8.213±0.9 \| \| --- \| | 9.011±2 |
| 4 | 1 | 13.742±1.3 | 14.069±1.9 |
| 5 | 2 | 20.559±3.2 | 18.472±0.5 |
| 6 | 3 | 28.379±3.0 | 23.797±1 |
| 7 | 4 | 39.309±2.5 | 36.138±2.3 |
| 8 | 6 | 52.673±1.6 | 43.958±1.5 |
| 9 | 12 | 66.855±0.8 | 54.253±3.5 |
| 10 | 24 | 74.880±2.3 | 65.919±1.8 |
| 11 | 48 | 77.258±1.8 | 79.386±2.1 |
| 12 | 72 | 80.495±3.1 | 88.536±3.8 |

*Table S3: Percentage of Cis released in phosphate buffer at pH 7.4 and 6.8 at specific time intervals for determination of best kinetic model*
