# Supplementary figures and images for: Green synthesis of hyaluronic acid coated, thiolated chitosan nanoparticles for CD44 targeted delivery and sustained release of Cisplatin in cervical carcinoma
Source: Front Pharmacol. 2023 Jan 12;13:1073004. doi: 10.3389/fphar.2022.1073004 (PMC9877355; doi:10.3389/fphar.2022.1073004)

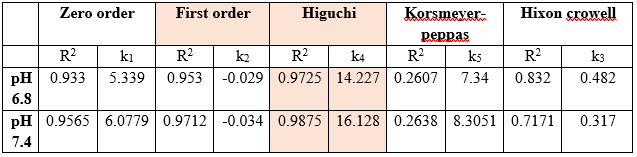


*Table S4: Kinetic models of drug release from NFs at pH 6.8 and 7.4*

Supplement: Supplementary file 5 [file Table4.docx]
